# The Ebola-Glycoprotein Modulates the Function of Natural Killer Cells

**DOI:** 10.3389/fimmu.2018.01428

**Published:** 2018-07-02

**Authors:** Avishay Edri, Avishai Shemesh, Muhammed Iraqi, Omri Matalon, Michael Brusilovsky, Uzi Hadad, Olga Radinsky, Orly Gershoni-Yahalom, John M. Dye, Ofer Mandelboim, Mira Barda-Saad, Leslie Lobel, Angel Porgador

**Affiliations:** ^1^The Shraga Segal Department of Microbiology, Immunology and Genetics, Faculty of Health Sciences, Ben-Gurion University of the Negev, Beer Sheva, Israel; ^2^National Institute for Biotechnology in the Negev, Ben-Gurion University of the Negev, Beer Sheva, Israel; ^3^The Mina and Everard Goodman Faculty of Life Sciences, Bar-Ilan University, Ramat-Gan, Israel; ^4^U.S. Army Medical Research Institute of Infectious Diseases, Frederick, MD, United States; ^5^The Lautenberg Center for General and Tumor Immunology, The BioMedical Research Institute Israel Canada of the Faculty of Medicine (IMRIC), The Hebrew University Hadassah Medical School, Jerusalem, Israel; ^6^Department of Emerging and Reemerging Diseases and Special Pathogens Uganda Virus Research Institute (UVRI), Entebbe, Uganda

**Keywords:** Ebola GP, Ebola, modulation of immune system, viral evasion strategies, NKG2D, MICA, steric shielding, natural killer cells

## Abstract

The Ebola virus (EBOV) uses evasion mechanisms that directly interfere with host T-cell antiviral responses. By steric shielding of human leukocyte antigen class-1, the Ebola glycoprotein (GP) blocks interaction with T-cell receptors (TCRs), thus rendering T cells unable to attack virus-infected cells. It is likely that this mechanism could promote increased natural killer (NK) cell activity against GP-expressing cells by preventing the engagement of NK inhibitory receptors; however, we found that primary human NK cells were less reactive to GP-expressing HEK293T cells. This was manifested as reduced cytokine secretion, a reduction in NK degranulation, and decreased lysis of GP-expressing target cells. We also demonstrated reduced recognition of GP-expressing cells by recombinant NKG2D and NKp30 receptors. In accordance, we showed a reduced monoclonal antibody-based staining of NKG2D and NKp30 ligands on GP-expressing target cells. Trypsin digestion of the membrane-associated GP led to a recovery of the recognition of membrane-associated NKG2D and NKp30 ligands. We further showed that membrane-associated GP did not shield recognition by KIR2DL receptors; in accordance, GP expression by target cells significantly perturbed signal transduction through activating, but not through inhibitory, receptors. Our results suggest a novel evasion mechanism employed by the EBOV to specifically avoid the NK cell immune response.

## Introduction

The Ebola virus (EBOV), a member of the Filoviridae family, is the cause of Ebola hemorrhagic fever. Throughout the past five decades, the five known species of this single-stranded, negative-sense RNA virus has caused sporadic outbreaks of Ebola fever in Central and West Africa, leading to high mortality in both humans and non-human primates. The virus is thus classified by the National Institutes of Health as a Category A select agent (1). Several structural proteins and one nonstructural protein are encoded in the EBOV genome. The structural proteins comprise a type I transmembrane glycoprotein (GP), a nucleoprotein (NP), four virion structural proteins (VP24, VP30, VP35, and VP40), and a RNA-dependent RNA polymerase (2). A number of studies investigating the expression of these proteins in cell culture have shown that the expression of EBOV-GP in cultured cells leads to a loss of cell–cell interaction and to cell rounding and detachment, and in cultures of endothelial cells, to detachment-mediated apoptosis (3–7). However, transient expression of EBOV-GP, by itself, did not seem to have a cytotoxic effect (4). Recent studies show that EBOV-GP shields some surface proteins in GP-expressing cells, particularly human leukocyte antigen class-I (HLA-I) molecules (8, 9)—a phenomenon that modulates the interactions between EBOV-infected cells and CD8 T lymphocytes (9). It is known that HLA-I molecules on target cells interact with receptors of natural killer (NK) cells and modulate NK function, but the direct effect of EBOV-GP on the interaction between NK cells and EBOV-GP expressing cells has never been tested.

Natural killer cells are lymphocytes of the innate immune system that are selectively cytotoxic to target cells, such as virus-infected cells and tumor cells. The cellular cytotoxicity of NK cells is delicately balanced by signals mediated through inhibitory and activating receptors. The expression of inhibitory ligands, such as HLA-I, which are recognized by killer-cell immunoglobulin-like receptors (KIRs), serves as a positive indicator of the integrity of the target cells, protecting them against NK-cell-mediated cytolysis (10, 11). In contrast, expression of activating ligands, which are usually stress-induced antigens that signal cellular distress caused by transformation or infection, could evoke an NK cell response. Such a response would induce both lysis of the expressing cells and cytokine secretion by the NK cells to “alarm” the immune system (12). It may thus be expected that the activity of NK cells would increase with the masking of HLA-I by EBOV-GP. Indeed, cell subtype prediction using messenger RNA expression patterns indicated that NK-cell populations increase in human patients who survive infection (13). Similarly, patients with fatal Ebola infection were characterized by a lower NK cell frequency as compared to patients with non-fatal infection; particularly, CD56neg NK cells were associated to non-fatal infections (14). Surprisingly, however, studies performed on baboons and cynomolgus macaques have indicated a steady decrease in NK cell activity and number of NK cells in the peripheral blood, respectively (15–17). Moreover, in a mouse-adaptive EBOV infection, depletion of NK cells did not result in a dramatic change in mouse survival, and NK92MI cells were not activated by EBOV. Nonetheless, treatment of infected mice with the VSVΔG/EBOV GP vaccine led to an NK-cell-mediated improvement in mouse survival and to activation of NK92MI cells (18).

However, despite the accumulated knowledge, as described above, nothing is currently known about the effect of EBOV-GP on the membrane expression of activating ligands for NK cell receptors or about the interaction of activating and inhibitory NK cell receptors with GP-expressing human cells. Viral evasion mechanisms are known to directly interfere with the functioning of NK cells either by downregulating the expression of activating ligands, such as NKG2D ligands, or by upregulating expression of inhibitory ligands, such as HLA-I (19–28). The impaired expression of HLA-I molecules in cells infected by various viruses or in tumor cells is a well-known mechanism for evading the T-cell response. Nonetheless, since HLA-I molecules are strong inhibitory ligands for NK cells, their downregulation results in a loss of balance between inhibitory and activating signals, thereby activating the specific cytotoxic effect of NK cells (20–24, 26, 28, 29).

As accumulating evidence suggests that a potent innate immune response is tightly linked to the containment of Ebola filoviral infection (16, 30–32), we aimed to explore the mechanism of the Ebola-GP-mediated effect on NK cell function. To this end, we transiently transfected HEK293T cells with EBOV-GP with the aim to investigate its effects on (i) expression by target cells of activating and inhibitory ligands for NK receptors and (ii) the function of NK cells exposed to GP-expressing target cells. We showed that EBOV-GP expression by target cells favors the axis reducing NK activation, while not perturbing the NK inhibition axis.

## Materials and Methods

### Ethics Statement

Primary NK cells were purified from peripheral blood of healthy, adult, volunteer donors, recruited by written informed consent, as approved by the Institutional Review Board Ben-Gurion University of the Negev (BGU).

### Cell Lines

HEK293T (ATCC CRL-3216) and MICA-transfected JEG3 (ATCC HTB-36) cell lines (33) were cultured as recommended by ATCC in DMEMx1 (Gibco, 41965-039) supplemented with 10% fetal calf serum (FCS) (Gibco, 12657-029), 1% l-glutamine (Biological Industries, 03-020-1A), 1% Pen-Strep (BI, 03-031-1B), 1% sodium pyruvate (BI, 03-042-1B), 1% MEM-Eagle (Biological Industries, 01-340-1B), and 1% HEPES 1M (Biological Industries, 03-025-1B).

### Constructs and Transfections

We employed a membrane-bound form (NC_006432.1) of an expression clone of Sudan virus (SUDV)-GP-Gulu (1-649) (34) [where Gulu is a strain of (SUDV), one of the five known viruses in the Ebolavirus genus]. This GP-Gulu membrane-bound form was cloned into pCAGGS plasmid backbone and also cloned into the pEGFP-N1 vector with the aim to obtain expression of green fluorescent protein (GFP)-fused GP upon transfection. We also used a full-length expression clone of the EBOV GP-Makona membrane-bound form, which was generously provided by Kartik Chandran, Albert Einstein College of Medicine, New York, NY, USA [the Makona variant of EBOV first appeared in the 2013 epidemic in West Africa (35, 36)]. To obtain GP or GP-GFP expressing cells, HEK293T cells were plated in 10-cm plates 24 h prior to transfection and transiently transfected using a calcium-phosphate-based reagent with 15 µg DNA per 10-cm plate. In all experiments floating cells were collected and pooled with Adherent Cells, harvested using a gentle non-enzymatic cell dissociation reagent (Versene, Gibco™, 15040-033), at 24 h post transfection.

### Antibodies, Fusion-Ig Proteins, and Reagents

The following antibodies (Abs) and other materials were used: phycoerythrin (PE)-conjugated anti-human HLA-A, B, C (W6/32, BioLegend), PE-conjugated anti-human MICA (159227, R&D Systems), PE-conjugated anti-CD107a (H4A3, SouthernBiotech), PE-conjugated anti-human IgG (polyclonal, Jackson ImmunoResearch), FITC-conjugated anti-KIR2DL2 (CH-L, BD Biosciences), FITC-conjugated anti-human IgG (polyclonal, Jackson ImmunoResearch), Alexa Fluor 647-conjugated streptavidin (Jackson ImmunoResearch), allophycocyanin (APC)-conjugated anti-mouse IgG (polyclonal, Jackson ImmunoResearch), Pacific Blue-conjugated anti-human CD16, purified/biotinylated anti-SUDV-GP (3C10), purified anti-B7H6, Capture: purified anti-human IFN-γ (NIB42, BioLegend), Detection: biotin anti-human IFN-γ (4S.B3, BioLegend), 7-aminoactinomycin D (7AAD) (BioLegend), anti-phosphotyrosine 4G10, anti-PLCγ1 (Upstate), anti-SHP-1 (Santa Cruz), anti-GAPDH (Biodesign), and *p*-nitrophenyl phosphate (pNPP; New England BioLabs). The following fusion-Igs were used as previously described; NKG2D-Ig (37), NKp30-Ig (38), NKp44-Ig (39), NKp46-Ig (40), KIR2DL4-Ig (41), KIR2DL1-Ig (27), and KIR2DL2-Ig (42).

### ELISA

To determine the interaction between EBOV-GP and fusion-Ig or anti-GP monoclonal antibody (mAb), ELISA plates were coated overnight at 4°C with 10 µg/ml of the recombinant GP proteins originating from Ebola Sudan. Blocking buffer (PBS + 2.5% skim milk) was applied for 2 h at room temperature, after which the plates were incubated with 2 µg/ml of fusion-Ig, anti EBOV-GP mAb, or PBS for 2 h at room temperature. Fusion-Ig was detected using horseradish peroxidase (HRP)-conjugated anti-human IgG (1 h at 1:1,000). SUDV-GP-Gulu 1-649 ectodomain was produced by expression in HEK293T cells with a HIS tag and purification of the secreted product by nickel column chromatography. For IFN-γ detection, 96 *U*-well plates were coated overnight with 100 μl/well of anti-human IFN-γ (capture mAb, 1 μg/well). Blocking (PBS + 10% FCS) was applied for 2 h at room temperature. Following 2 h of incubation with IFN-γ-containing medium, biotin anti IFN-γ detection mAb (2 μg/well) was added to each well. For detection, streptavidin-HRP (Jackson, 016-030-084) diluted 1:1,000 was added for 30 min. Following washing, TMB (DAKO, S1599) was added to each well, and optical density (O.D.) was read at 650 nm (Thermo Electron Corporation Multiskan Spectrum). Between each step, wells were washed three times with PBS containing 0.05% Tween 20 (PBST).

### Trypsin Treatment

At 24 h post-transfection, Adherent and floating Cells were harvested, pooled, washed with PBS, and incubated in 1 ml trypsin EDTA solution (Biological industries, 03-052-1B) for 2.5 min on ice. Trypsin activity was stopped by addition of 7 ml ice-cold complete DMEM growth medium supplemented with 10% FCS. Cells were then centrifuged and supernatant was discarded.

### Flow Cytometry

Flow cytometry was employed for analysis of cell surface marker expression. Adherent and floating Cells were harvested, pooled, and plated 10^5^ cells/well. Cells were incubated on ice for 45 min with various mAbs and fusion-Igs (100 µg/ml, 50 µl/well). Fusion-Ig was detected with APC- or PE-conjugated anti-human-IgG Ab. Dead cells were detected with 7AAD. Flow cytometry was performed with a FACSCanto II (BD Biosciences), and results were analyzed using FlowJo^®^ software (Tree Star).

### Primary NK Cell Purification

Cells were isolated using a human negative selection-based NK isolation kit (RosetteSep, Miltenyi Biotec). Purified NK cells were then cultured in stem cell serum-free growth medium (CellGenix GMP SCGM, 20802-0500) supplemented with 10% heat-inactivated human AB plasma from healthy donors (SIGMA, male AB, H-4522), 1%l-glutamine, 1% Pen-Strep, 1% sodium pyruvate, 1% MEM-Eagle, 1% HEPES 1M, and 300 IU/ml recombinant human IL-2 (PeproTech).

### IFN-γ Secretion, CD107a Degranulation, and Lysis Assays for NK Cell Activity

For IFN-γ and CD107a degranulation assays, primary NK effector cells (5E4 cells/well) were mixed with target cells (1.5E5 cells/well) and incubated at 37°C for 18 or 5 h, respectively. For lysis assays, target cells were labeled with carboxyfluorescein succinimidyl ester (CFSE) (Life Technologies, C34554) and incubated with IL-2-activated primary NK cells at various ratios for 5 h at 37°C. Dead cells were detected using 7AAD by flow cytometry (Canto-II, BD Biosciences), and data were analyzed by FlowJo. Specific lysis (%) was calculated as previously described (43).

### NK Cell Activation by Adherent Proteins

Wells (96 U plate) were coated with anti-NKp44 activating mAb, soluble recombinant SUDV-GP, medium preconditioned with GP-expressing cells (“sup”), or PBS and further incubated with pNK cells in the presence of 25 U/ml rhIL-2 at 37°C for 18 h. The culture media were then sampled, and IFNγ was quantified using an ELISA assay.

### PTP Assay

SH2-domain-containing protein tyrosine phosphatase-1 catalytic activity was determined by measuring the hydrolysis of its exogenous substrate pNPP, as previously described (44). NK cells (10 × 10^6^) were incubated with target cells at ratio of 1:1 at 37°C for 5 min before lysis. Cells were lysed with passive ice-cold lysis buffer [1.25% Brij^®^, 0.625% *n*-octyl-β-d-glucoside, 31.3 mM Tris–HCl, pH 7.4, 150 mM NaCl, 6.25 mM EDTA, and complete protease inhibitor tablets (Roche)]. Where indicated, 2 mM pervanadate was added to the lysis buffer as a negative control. Cell lysates were subjected to immunoprecipitation (IP) with anti-SHP-1 antibody (Santa Cruz). Immunoprecipitates were washed twice with ice-cold passive washing buffer (0.1% Brij, 50 mM Tris–HCl, pH 7.4, 300 mM NaCl, and 3.75 mM EDTA) and three times with phosphatase buffer (150 mM NaCl, 50 mM HEPES, 10 mM EDTA, and 1 mM DTT). Immunoprecipitates were resuspended in 200 µl of 50 mM pNPP in phosphatase buffer and incubated for 30 min at 37°C. Reactions were terminated by adding 800 µl of 1 M NaOH, and SHP-1 activity was determined by measuring the absorbance at 405 nm. The results were normalized to the amount of precipitated SHP-1 and are presented relative to the activity measured in NK cells incubated with mock transfected HEK293T cells.

### Cell Stimulation, Immunoblotting, and IP

Natural killer cells and target cells were first incubated separately on ice for 10 min, at a ratio of 1:1. The cells were then mixed, centrifuged, and kept on ice for 15 min. Thereafter, the cell mixture was held at 37°C for 5 min and then lysed with ice-cold lysis buffer (1.25% Brij, 0.625% *n*-octyl-β-d-glucoside, 31.3 mM Tris–HCl, pH 8, 150 mM NaCl, 6.25 mM EDTA, 1.25 mM Na_3_VO_4_, and complete protease inhibitor tablets).

For analysis of whole cell lysates or IP experiments, 5 × 10^5^ cells or 5 × 10^6^ cells were used, respectively. Protein A/G plus-agarose beads (Santa Cruz Biotechnology) were used for IP. Protein samples were resolved by sodium dodecyl sulfate-polyacrylamide gel electrophoresis (SDS-PAGE), transferred to nitrocellulose membranes and immunoblotted with the appropriate primary antibodies. Immunoreactive proteins were detected with either anti-mouse or anti-rabbit HRP-coupled secondary antibody, followed by detection by enhanced chemiluminescence (PerkinElmer).

### Statistical Analysis

Graphics and statistical analysis were performed using Microsoft Office/Excel software. Statistical analysis of the data was performed using Student’s unpaired two-tailed *t*-test (with *p*-values of <0.05, <0.01, or <0.001, as indicated on the figures).

## Results

**Figure 1:**
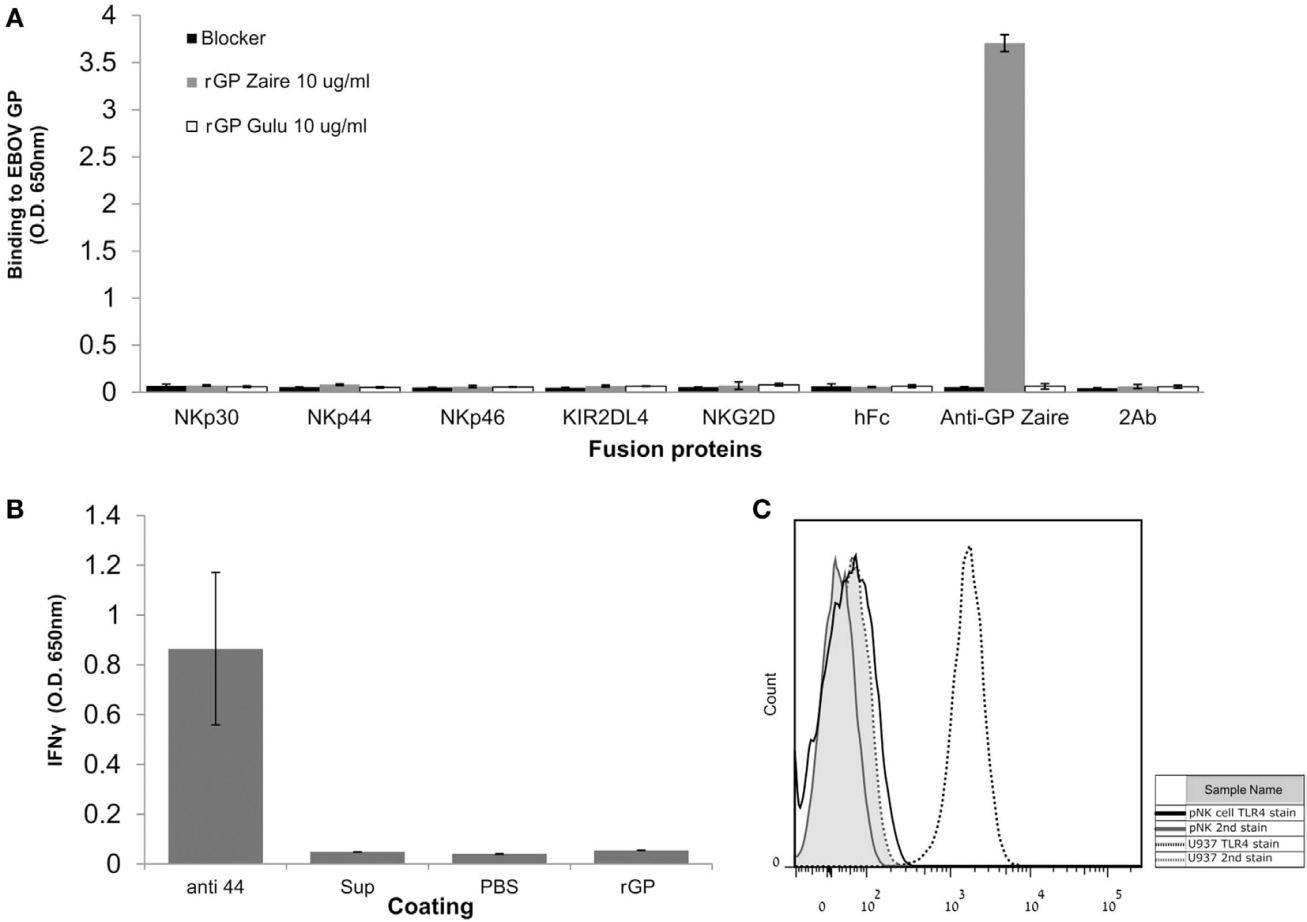
Natural killer (NK)-cell-expressed receptors do not interact directly with EBOV-GP. **(A)** ELISA plates were coated with either soluble recombinant Sudan virus (SUDV)-GP (Sudan ebolavirus GP) or Zaire ebolavirus GP and incubated with fusion-Ig, anti EBOV-GP monoclonal antibody (mAb), or PBS to assay for direct interaction. Fusion-Ig was detected with horseradish peroxidase-conjugated anti-human IgG. **(B)** Plastic wells were coated with either anti-NKp44 activating mAb, soluble recombinant SUDV-GP, medium preconditioned with GP-expressing cells (“sup”), or PBS and incubated with pNK cells in the presence of 25 U/ml rhIL-2. The culture media were sampled, and IFNγ was quantified using an ELISA assay. **(C)** Primary NK was stained with phycoerythrin-conjugated anti-human TLR4. As a positive biological control, U937 cells that express TLR4 were stained too. Results are from one representative experiment of three **(A)** and two **(B,C)** performed. Values represent means of triplicates; Bars, ±SD.

We and others have found that different viral GPs can be recognized by NK receptors (40). Therefore, we first investigated whether the Ebola GP interacts directly with different NK receptors. Gulu is a strain of SUDV, one of the five known viruses in the Ebolavirus genus. We employed soluble recombinant SUDV-GP (rSUDV-GP) of the Gulu strain. rSUDV-GP did not interact directly with a panel of NK receptors, including members of the KIR2DL family, the NCR family, and the NKG2D receptor (Figure 1A). Upon incubating NK cells in SUDV-GP coated wells, we did not observe any changes in NK-mediated IFNγ secretion (Figure 1B). This observation is in accordance with previous reports (18), suggesting that the GP proteins do not engage NK receptors tested herein (NKp30, NKp44, NKp46, KIR2DL4, NKG2D) and, therefore, they modulate NK activity *via* another mechanism. TLR4 was reported to bind to GP, but TLR4 expression in NK is reported to be restricted to the intracellular location (45–48). We verified that indeed in our primary NKs, TLR4 is not expressed on the cell membrane (Figure 1C), and thus TLR4 cannot contribute to recognition of Ebola GP by NK.

### Surface Expression of Different Ebola GPs Reduces Membrane-Associated Staining of HLA-I and of Ligands to the NK-Activating Receptors, NKG2D and NKp30

**Figure 2:**
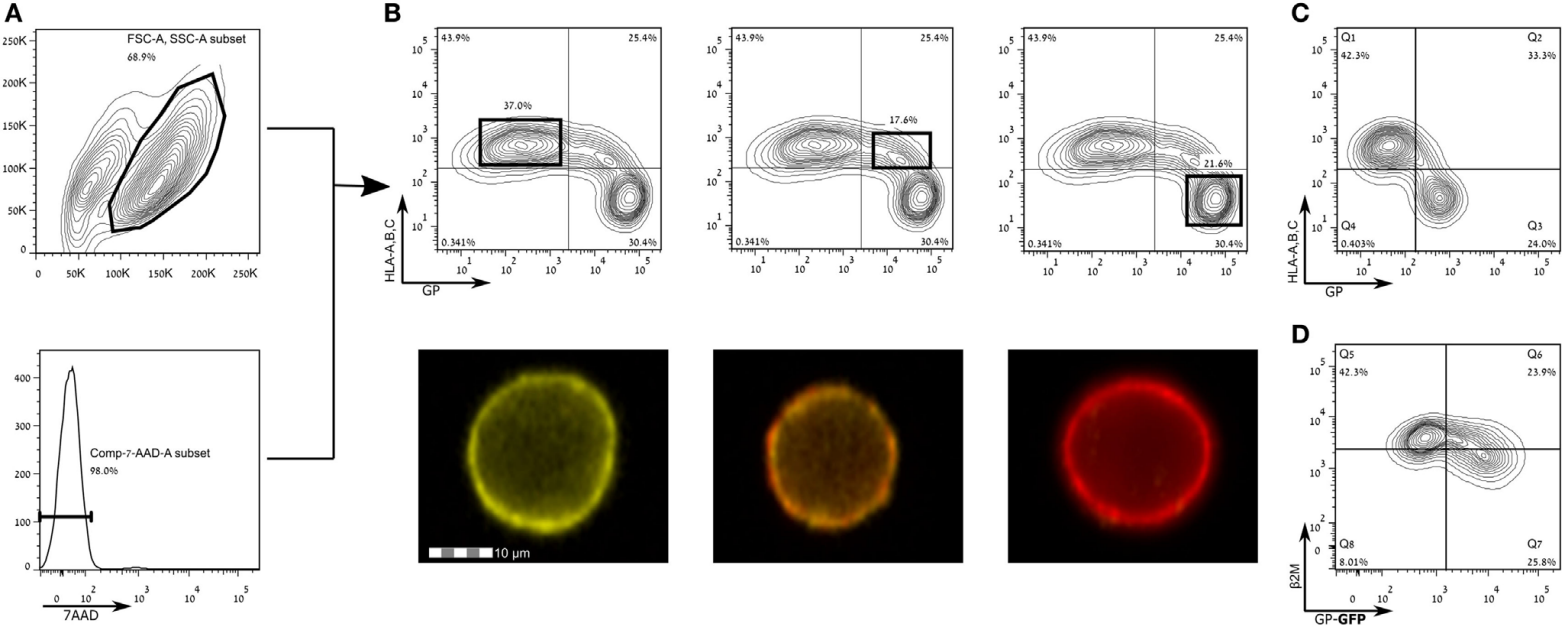
Expression of Sudan virus (SUDV)- and Makona-GPs results in loss of surface staining of HLA-A, B, C. The figure shows flow cytometry analysis of steric shielding of HLA-A, B, C antigens by Ebola GP. **(A)** SUDV-GP transfected HEK293T cells were harvested using a non-enzymatic reagent, and dead cells were excluded by 7-aminoactinomycin D. **(B)** Cells were stained for SUDV-GP with a biotinylated 3C10 antibody, followed by allophycocyanin-conjugated streptavidin, and co-stained for HLA-A, B, C surface antigens with a phycoerythrin-conjugated W6/32 antibody. Representative examples are shown in image stream fluorescent microscopy (Yellow: HLA-A, B, C. Red: SUDV-GP). **(C)** Makona-GP-transfected cells stained for expression of Makona-GP and co-stained for HLA-A, B, C surface antigens. **(D)** SUDV-GP-GFP transfected cells were stained for β2 microglobulin. Results for **(C,D)** are from one representative experiment of two performed. Dot plot results shown in **(B)** are from one representative analysis of 30 independent flow cytometry analyses.

Francica et al. found that expression of EBOV-Zaire GP results in a loss of cell surface staining of HLA-I (9). Alazard-Dany et al. showed that infection of HEK293T cells with recombinant EBOV subtype Zaire strain Mayinga resulted in the same observation of loss of cell surface staining of HLA I (49). Zaire ebolavirus is the most pathogenic subtype in humans (50, 51), and thus we aimed to test whether GPs from other, less pathogenic EBOV strains, namely, SUDV-Gulu and the Makona strains of EBOV, are also responsible for mediating this phenomenon. To this end, we expressed the membrane-bound form of GPs of SUDV and the Makona strain in HEK293T cells and stained for HLA-I expression. Figures 2A,B shows that HLA-I staining was reduced in live gated HEK293T cells expressing high levels of SUDV-GP, whereas in low- to moderate-GP expressers, there was no reduction in HLA-I staining. Image stream analysis of live cells showed the corresponding reduction (or lack thereof) in staining (Figure 2B, bottom insets). Similar to SUDV-GP, high Makona-GP expressers significantly reduced staining of HLA-I (Figure 2C). We verified this result with an antibody specific to human β_2_ microglobulin (Figure 2D). Note that HLA-I staining in mock transfected cells was similar to HLA-I staining of low-GP expressers (Figure S1 in Supplementary Material). The GP-high mediated significant reduction of cell surface HLA-I could not be attributed to overall reduction in cellular HLA-I expression since intracellular staining for HLA-I revealed similar expression levels between high and low GP expressers (Figures S2A,B in Supplementary Material).

**Figure 3:**
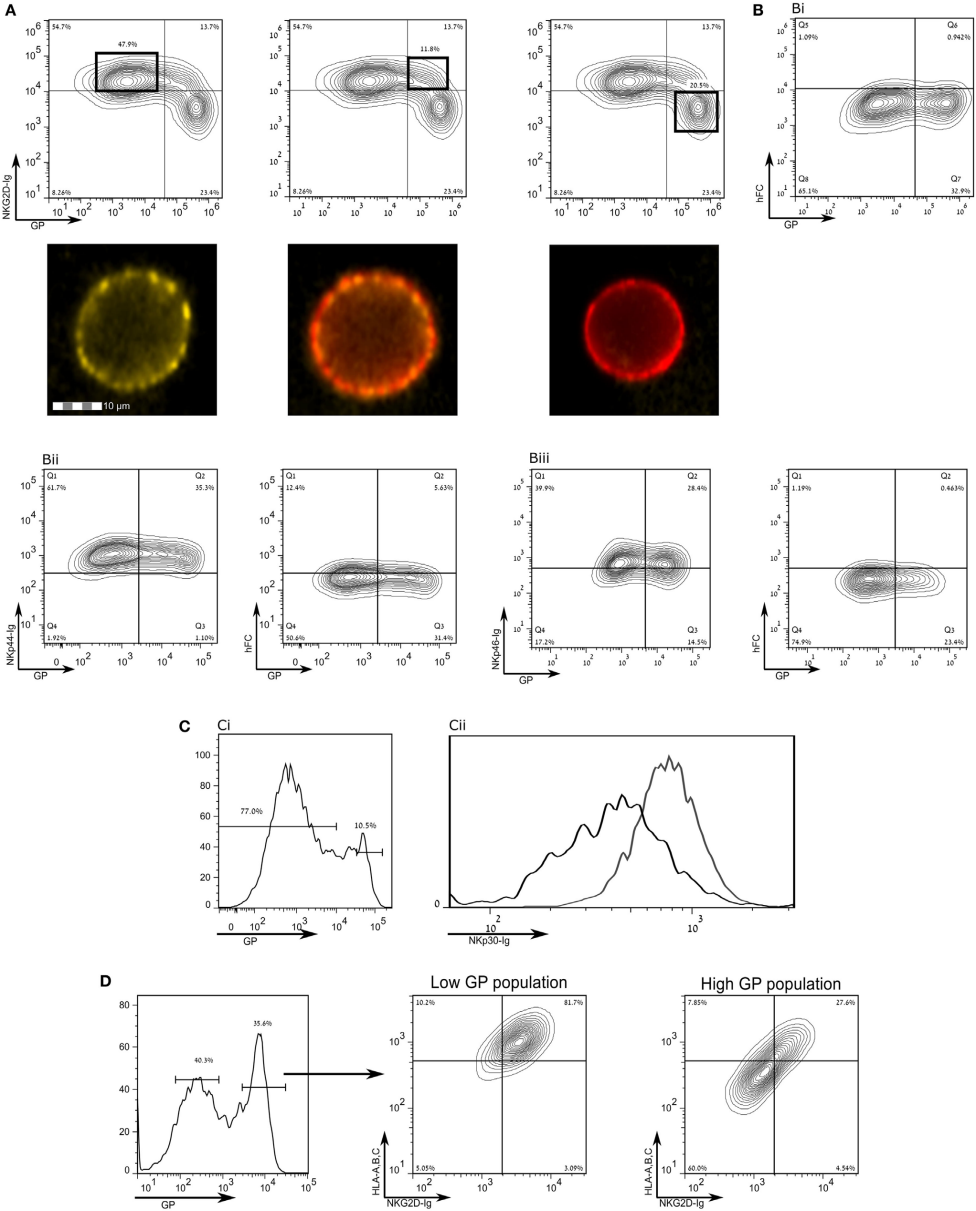
Expression of the Sudan virus (SUDV)-glycoprotein (GP) results in a loss of surface staining of ligands for natural killer cell receptors. The figure shows flow cytometry analysis of steric shielding of membrane antigens by SUDV-GP. **(A)** SUDV-GP-transfected HEK293T cells were stained for SUDV-GP and co-stained for NKG2D ligands with an NKG2D-Ig chimeric protein, followed by phycoerythrin-labeled secondary antibody. Representative examples are shown in image stream fluorescent microscopy (Yellow: NKG2D-Ig. Red: SUDV-GP). **(B)** SUDV-GP-transfected HEK293T cells were stained with anti-SUDV-GP and with: **(Bi)** purified human Fc to determine the background of NKG2D-Ig staining [panel **(A)** above]; **(Bii)** an NKp44-Ig chimeric protein to stain for NKp44 ligands [left panel of **(Bii)**]. Background was determined once again by staining with purified human Fc [right panel of **(Bii)**]; **(Biii)** an NKp46-Ig chimeric protein to stain for NKp46 ligands [left panel of **(Biii)**]. Background was determined once again by staining with purified human Fc [right panel of **(Biii)**]. **(C)** Cells were stained for NKp30 ligands with an NKp30-Ig chimeric protein and co stained for SUDV-GP, showing: **(Ci)** high- versus low-GP-expressing HEK293T cells; **(Cii)** plot of the binding of NKp30-Ig to high GP-expressing HEK293T cells (black line) and low GP-expressing HEK293T cells (gray line). **(D)** SUDV-GP-transfected HEK293T cells were stained for SUDV-GP and co-stained for HLA-A, B, C, and NKG2D ligands. Analysis shows high SUDV-GP expressers gated versus low SUDV-GP expressers analyzed for staining of both HLA-A, B, C, and NKG2D ligands. Results are from one representative experiment of 10 flow cytometry and two ImageStream experiments performed.

Human leukocyte antigen class-1 presents peptides to T-cell receptors (TCRs) and also serves as a ligand for NK receptors, mostly inhibitory type receptors. Yet, in apparent contradiction, EBOV infection is associated with a reduction of NK activity in infected monkeys (15–17). We, therefore, investigated whether staining for activating ligands of NK cell receptors in SUDV-GP- and Makona-GP-transfected HEK293T cells is reduced following GP expression in those cells. We employed recombinant NK-activating receptors to cover numerous ligands recognized by NK cells. Staining with NKG2D-Ig was significantly reduced following high GP expression (Figure 3A), as confirmed by image stream analysis of live cells (Figure 3A, bottom insets). Note that staining with NKG2D-Ig for high GP expressers was suppressed to the level of staining with control human Fc (Figures 3A,Bi). Expression of GP had no effect on staining with control human Ig (Figure 3Bi) or with recombinant NKp44-Ig and NKp46-Ig (Figures 3B**ii**,**iii**, respectively; including the corresponding staining with human Fc control). GP expression did, however, affect staining with NKp30-Ig, but only to a moderate extent (Figure 3C). To test whether the marked reduction of staining by NKG2D-Ig and anti-HLA-I was in the same high GP expresser cells population, we triple-stained for GP, HLA-I, and NKG2D (Figure 3D); high-GP expressers reduced staining both of HLA-I and NKG2D ligands, as compared to low- to moderate-GP expressers. Note that NKG2D-Ig and NKp30-Ig staining of mock transfected cells was similar to NKG2D-Ig and NKp30-Ig staining of low-GP expressers (Figure S1 in Supplementary Material).

**Figure 4:**
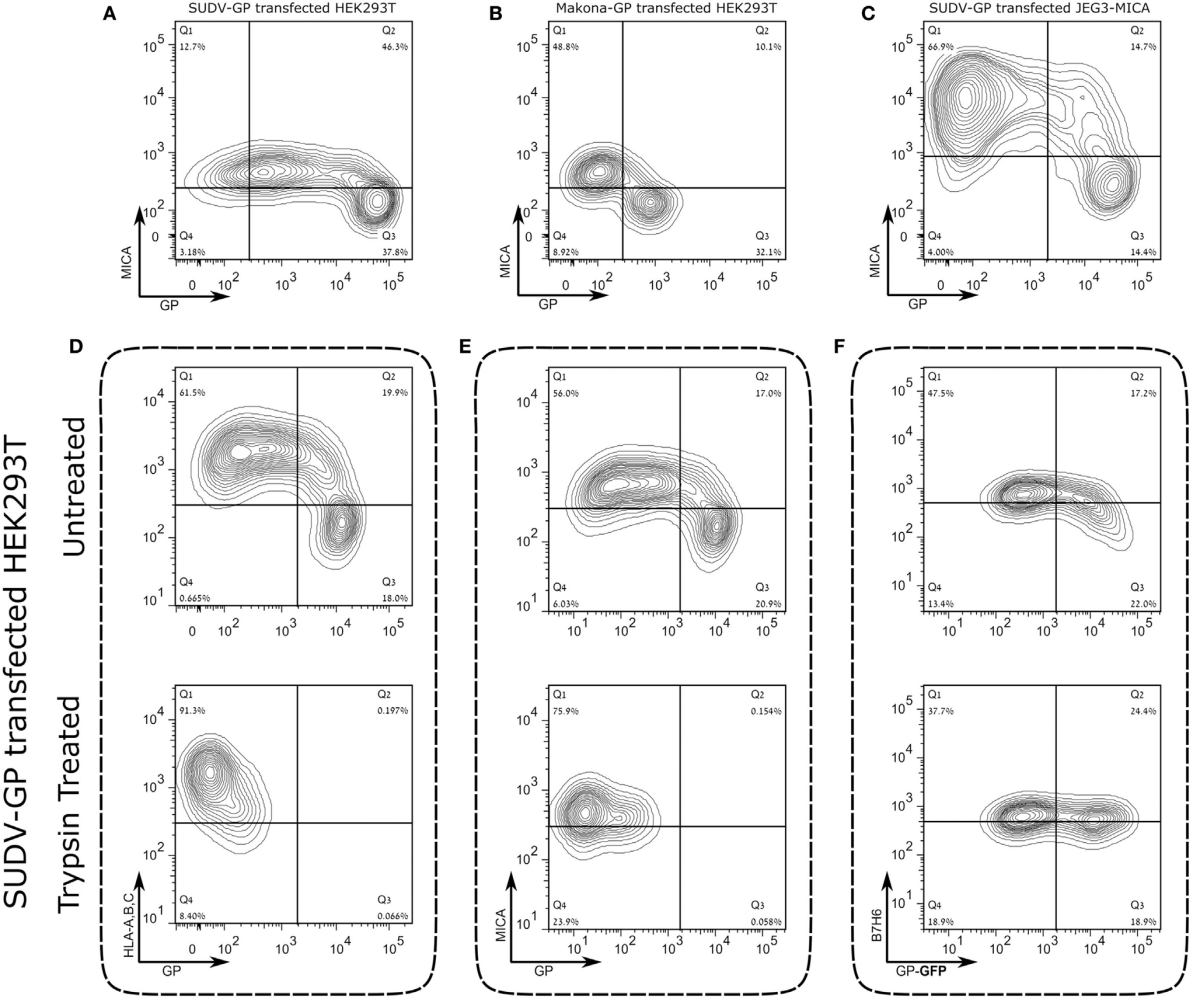
Expression of the Sudan virus (SUDV)- or Makona-GP results in loss of surface staining of αMICA in HEK293T cells and JEG3 cells stably transfected with MICA. The figure shows representative flow cytometry analysis for steric shielding of the MICA antigen by SUDV-GP or Makona-GP. **(A)** HEK293T cells were transfected, harvested, and stained for SUDV-GP with a biotinylated 3C10 antibody, followed by allophycocyanin (APC)-conjugated streptavidin, and co-stained for MICA surface antigens with phycoerythrin (PE)-conjugated αMICA antibody. Results are from one representative experiment of more than 10 performed **(B)** HEK293T cells were transfected, harvested, and stained for Makona-GP with survivor sera, followed by APC-labeled secondary antibody, and co-stained for MICA surface antigen using PE-conjugated αMICA antibody. Results are from one representative experiment of two performed. **(C)** SUDV-GP-transfected JEG3-MICA cells stained for SUDV-GP and co-stained for MICA surface antigens and NKG2D-Ig ligands. Results are from one representative experiment of more than four performed. **(D–F)** HEK293T cells were transfected with SUDV-GP or SUDV-GP-GFP, harvested shortly after they were treated with trypsin or left untreated, and finally stained for: **(D)** HLA-A, B, C, and SUDV-GP, **(E)** MICA and SUDV-GP, and **(F)** B7H6. Results are from one representative experiment of more than four performed.

To verify our observations with Abs specific for NKG2D ligands expressed by cells, we first stained with antibody to MICA, which is expressed by HEK293T cells. MICA staining was significantly reduced following high SUDV-GP expression by HEK293T cells (Figure 4A). Similarly, high Makona-GP expression resulted in elimination of MICA staining on HEK293T cells (Figure 4B). We also repeated these experiments in MICA-stably transfected JEG3 cells (JEG3-MICA), which were subsequently transfected with SUDV-GP. MICA staining of high GP-expressers-MICA-JEG3 cells was significantly reduced (Figure 4C). Note that MICA staining in mock transfected cells was similar to MICA staining of low-GP expressers (Figure S1 in Supplementary Material). As we observed for HLA-I, the GP-high mediated significant reduction of cell surface MICA could not be attributed to overall reduction in cellular MICA expression since intracellular staining for MICA revealed similar expression levels between high and low GP expressers (Figures S2C,D in Supplementary Material). Moreover, we further tagged MICA with fused fluorophore; similarly to Figure S2D in Supplementary Material, we observed that FL-tagged MICA expression, representing MICA levels within the cell and on the membrane did not change between high and low GP expressers (Figure S2E in Supplementary Material).

We further assessed whether the significant reduction of NKG2D and NKP30 ligands associated with high GP expression could be observed for additional viral GP. We investigated influenza virus H5-expressing cells that manifested low and high H5 expression and did not observe any effect induced by high H5 phenotype on staining with NKG2D-Ig and NKp30-Ig (Figures S2F–I in Supplementary Material).

### Staining of Membrane-Associated HLA-I, NKG2D Ligands, and NKp30 Ligands Is Restored by Trypsin Treatment That Cleaves Surface Ebola-GP

A critically important observation of Francica et al. was that HLA-I is not deleted from the cell surface following GP expression, but, rather, it is shielded from Ab-mediated staining by the expressed GP (9) [the authors employed DTT treatment to expose the GP-masked cell-surface HLA-I (52)]. In a similar vein, we observed that cell surface GP expression on transfected HEK293T cells was very sensitive to trypsin treatment, whereas on untransfected HEK293T cells, the same trypsin treatment protocol had a less marked effect on membrane-associated expression of HLA-I, MICA, and the NKp30 ligand, B7-H6 (which is also expressed by HEK293T cells), as is shown in Figure S3A in Supplementary Material. Therefore, we tested whether a short trypsin treatment of GP-expressing HEK293T cells would reveal cell surface molecules that are shielded by the GP. Indeed, the short trypsin treatment (2.5 min) of GP-expressing cells restored HLA-I and MICA staining in GP-transfected HEK293T cells (Figures 4D,E; upper versus lower panels). To assess whether B7-H6 cell-membrane expression would also be restored following the removal of GP with a short trypsin treatment, we transfected HEK293T cells with GFP-fused SUDV-GP. Figure 4F (upper panel) shows that high GFP expressers reduced B7-H6 staining, but that the short trypsin treatment restored cell surface expression of B7-H6. Note that since GFP is fused to the cytoplasmic part of GP, trypsin treatment that removes the extracellular portion of GP does not affect the GFP signal (Figure 4F, lower panel). We further verified the results from our trypsin-based approach to degrade the ectodomains of GP with the DTT approach employed by Francica et al.; Figure S3B in Supplementary Material shows HLA-I/MICA versus GP staining with and without DTT treatment. Results were similar to the trypsin treatment showing that DTT treatment restored membrane-associated staining of HLA-I and MICA in the GP-transfected high-expressers. We developed the trypsin approach over the DTT approach to further assess NK functions; DTT treatment induces cell death in fraction of the treated target cells, while trypsin treatment does not. However, following trypsin treatment, cell surface GP expression recovered considerably within 1 h and in accordance GP-mediated shielding of cell surface HLA-I was restored (Figure S3C in Supplementary Material); thus, we could not perform NK function assays (described below) on trypsin-treated cells.

### SUDV-GP-Induced Disruption of Target Cell Antigens Downmodulates the Function of Primary Human NK

**Figure 5:**
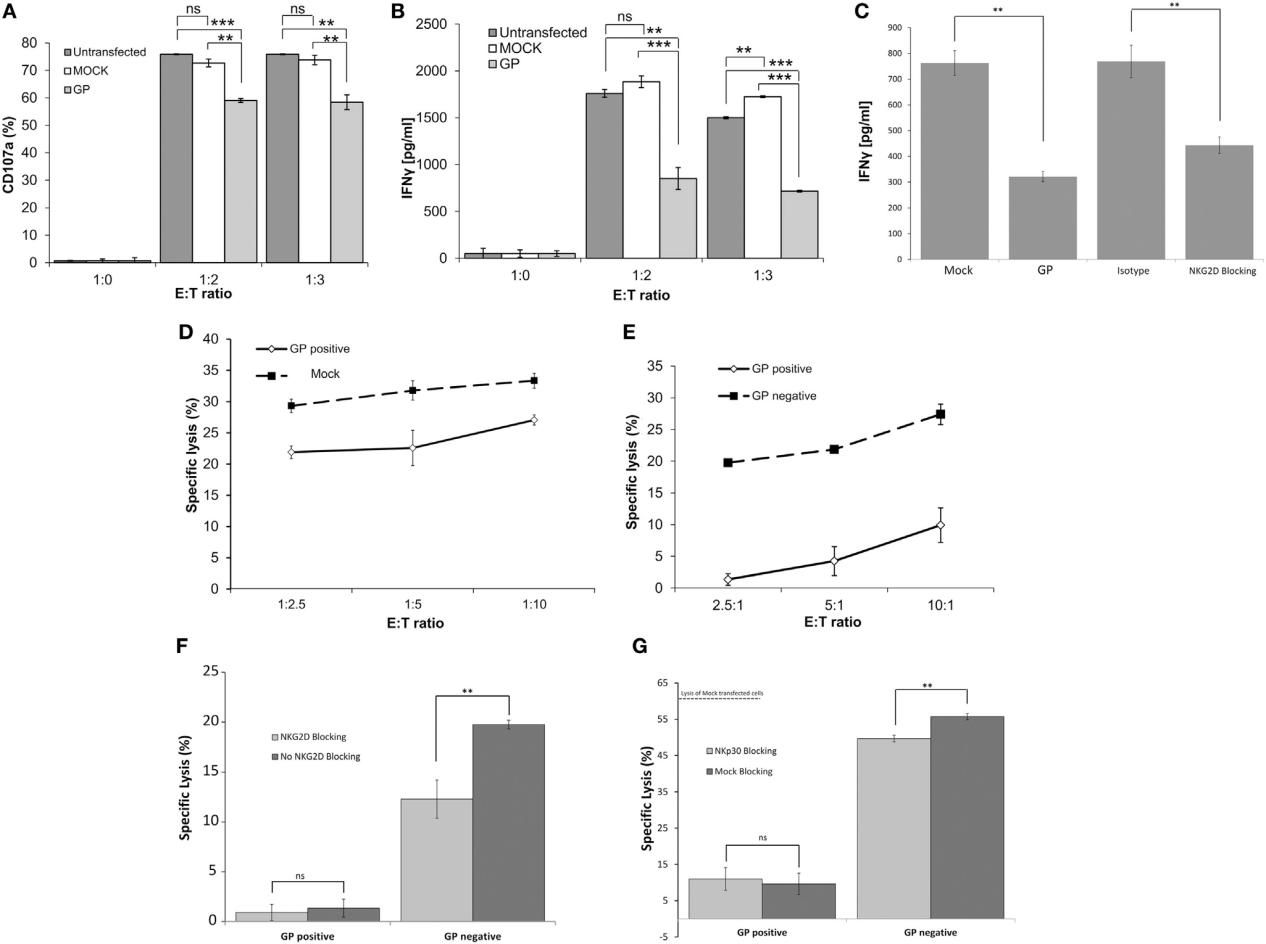
Sudan virus (SUDV)-GP-induced disruption of surface antigens downmodulates pNK activation. HEK293T cells were either SUDV-GP transfected, mock transfected, or left untransfected and cocultured with pNK cells in the presence of 25 U/ml rhIL2. Effector cells, target cells, and growth media were sampled and assayed for natural killer cell activation markers. **(A)** Target and effector cells were cocultured at the ratios specified in the figure for 6 h. Cells were then harvested and analyzed for degranulation by flow cytometry. Dead cells were excluded by 7-aminoactinomycin D (7AAD); pNK cells were then gated by staining for CD16 and co-stained for degranulation by staining for CD107a. **(B)** Target and effector cells were cocultured at the specified ratios for 18 h. The culture medium was sampled, and IFNγ was quantified using an ELISA assay. **(C)** Target cells were either SUDV-GP transfected or Mock-transfected; Mock-transfected cells were then incubated with anti-NKG2D or incubated with isotype control and cocultured with effector cells for 18 h. The culture medium was sampled, and IFNγ was quantified using an ELISA assay. **(D)** Carboxyfluorescein succinimidyl ester (CFSE)-stained Mock- or GP-transfected cells and unstained effector cells were cocultured at the specified ratios for 6 h. Cells were then harvested and analyzed for specific lysis by assessing the viability CFSE^+^ cells using 7AAD. **(E)** CFSE-stained GP-transfected target cells and unstained effector cells were cocultured at the specified ratios for 6 h. Cells were then harvested and analyzed for specific lysis by flow cytometry. Cells were stained for SUDV-GP with biotinylated 3C10 antibody followed by APC-conjugated streptavidin. CFSE^+^GP^+^ and CFSE^+^GP^−^ cells were gated and analyzed for viability using 7AAD. **(F)** Effect of anti-NKG2D blocking on specific lysis of GP-negative and GP-positive HEK293T cells by pNK cells (E:T 3:1). **(G)** Effect of blocking with anti-NKp30 versus incubation with isotype control on specific lysis of GP-negative and GP-positive HEK293T cells by pNK cells (E:T 3:1). Results are from one representative experiment of four **(A,B,D,E)** and two **(C,F)** performed. Values represent means of triplicates. Bars, ±SD. Student’s unpaired *t*-test; **p < 0.01; ***p < 0.001; ns, not significant.

To determine the degree of activation of NK cells exposed concomitantly or sequentially to different target cell types, we examined the expression of cell surface CD107a and IFNγ—as indicators of NK cell activation—after co-incubation of primary NK cells with heterogeneous target cells. The membrane-associated CD107a staining assay provides a measure of the degranulation of NK cells; the gating strategy is described in Figure S4A in Supplementary Material. Co-incubation of primary NK cells with non-treated HEK293T or mock-transfected HEK293T targets induced substantial and similar cell surface expression of CD107a. Co-incubation of primary NK cells with GP-HEK293T cells upregulated cell-surface CD107a, yet, to levels that were significantly reduced as compared to non-treated/mock-transfected target cells (Figure 5A). This NK-CD107a effect, mediated by GP transfection of target cells, was observed for NK obtained from different donors in repeated experiments (Figure S5A in Supplementary Material). An examination of NK-IFNγ secretion revealed that, in accordance with the CD107a results, NK cells secreted significantly less IFNγ when incubated with a GP-transfected target population compared with mock-transfected cells (Figure 5B). Note that NKG2D stimulation was imperative to IFNγ secretion by NK cells incubated with by mock transfected cells; blocking with anti-NKG2D mAb significantly reduced IFNγ secretion, as did GP transfection (Figure 5C). Similarly to CD107a, this IFNγ suppression by NK, facilitated by GP transfection of target cells, was repetitive with NK obtained from different donors (Figure S5B in Supplementary Material).

A lysis assay was used to specifically correlate target cell sensitivity to lysis by NK cells with the level of GP expression in whole GP-transfected cells versus mock-transfected cells. The gating strategy for the 7AAD-based lysis assay is described in Figure S4B in Supplementary Material. As was found in the CD107a assay, there was a statistically significant reduction in the lysis of GP-transfected cells; yet, the reduction in lysis did not surpass 25% even at an effector-to-target cell (E:T) ratio of 1:2.5 (Figure 5D comparing the lysis of mock- versus GP-transfected target cells). However, when we stained target GP-transfected cells for both GP and lysis markers, we could differentiate between the sensitivity to lysis of the different GP-expressing cell types: in the same GP-transfected population exposed to NK cells, the null/low-GP expressing target cells were lysed by NK cells, while the high GP-expressing target cells were resistant to lysis (Figure 5E). Here, it is important to note that we could perform this comparison of sensitivity to lysis of GP-positive and GP-negative target cells because the GP protein does not leak from the dead cells (as does GFP).

Since we found that the target cell ligands to the activating NKG2D receptor were significantly shielded by Ebola GP expression (Figures 2–4), we set out to compare the effect of blocking NK activity with antibody to NKG2D with the effect of GP expression on target cells. As predicted from the literature, blocking primary human NK cells with anti-NKG2D mAb significantly reduced the lysis by null/low GP expressers (Figure 5F). Yet, blocking with anti-NKG2D did not significantly affect the residual lysis of the GP-positive target cells (Figure 5F). An important finding was that GP expression on target cells resulted in a reduction in NK-mediated lysis that was substantially greater than the reduction obtained by blocking the NKG2D receptor and interacting the NK cells with GP-negative target cells. This finding suggests that GP masks not only NKG2D ligands but also other activating ligands, such as B7-H6 (Figure 4F). Indeed, blocking with anti-NKp30 mAb, as compared to blocking with isotype control, resulted in a low but significant reduction of lysis of target GP-negative transfected cells, while the residual lysis of GP-positive transfected cells was not affected from blocking with anti-NKp30 mAb (Figure 5G).

### GP Protein Downregulates NK Cell Function Through Inhibition of Activating Signals Rather Than Through the Induction of Inhibitory Cascades

**Figure 6:**
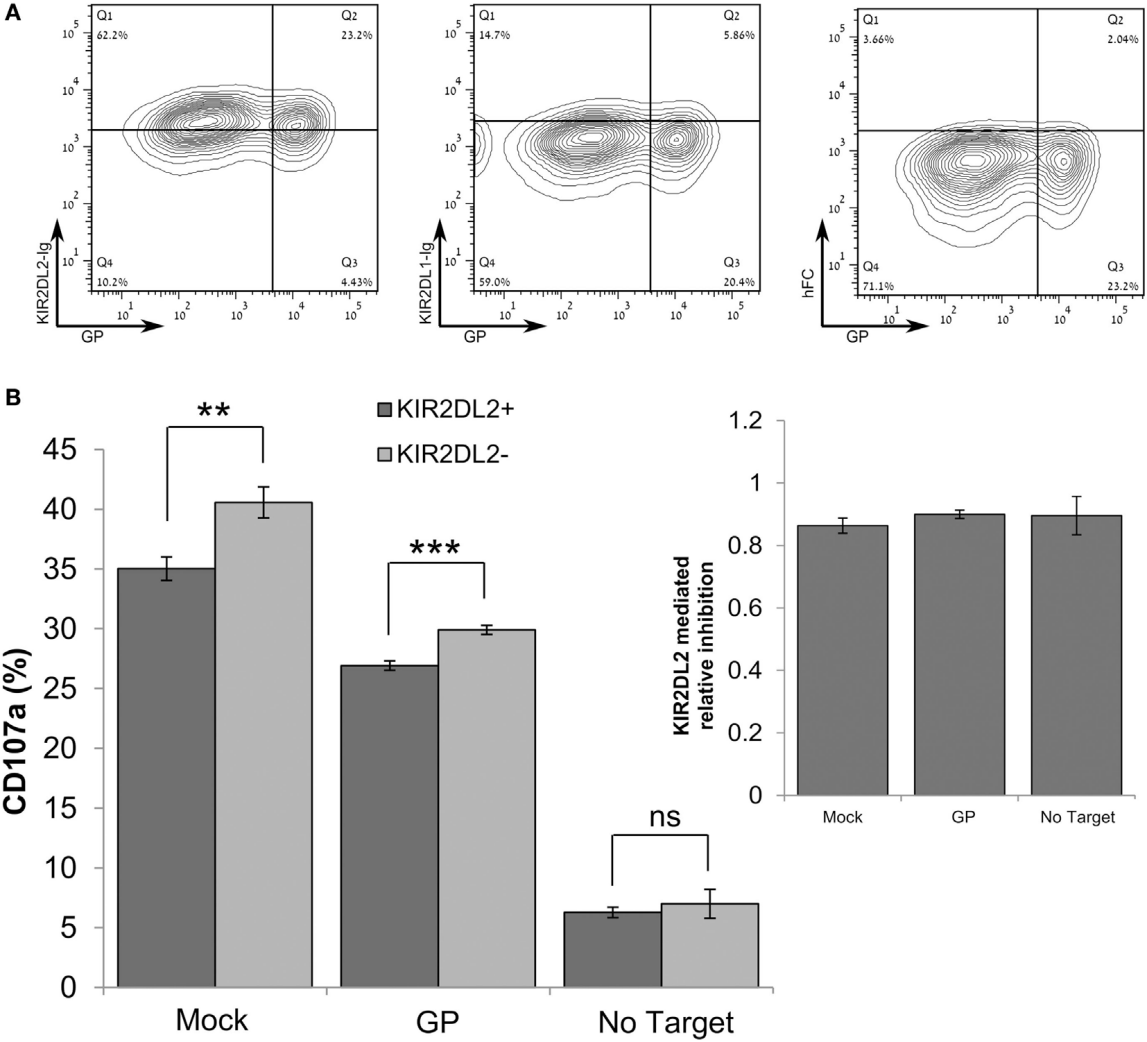
Sudan virus (SUDV)-GP-induced disruption of surface antigens does not affect KIR2DL2-mediated inhibitory pathway. In these experiments, HEK293T cells were SUDV-GP-transfected, mock transfected, or left untransfected. **(A)** SUDV-GP-transfected HEK293T cells were stained for SUDV-GP and co-stained for KIR2DL2 or KIR2DL1 ligands using KIR2DL2-Ig or KIR2DL1-Ig chimeric proteins, followed by PE-labeled secondary antibody. Background staining was determined by staining with purified human FC. **(B)** Target cells were cocultured with pNK cells in the presence of 25 U/ml rhIL-2. Target and effector cells were cocultured at an E:T ratio of 1:3 for 6 h. Cells were then harvested and analyzed for degranulation by flow cytometry. Dead cells were excluded by 7-aminoactinomycin D; pNK cells were then gated by staining for CD16 and KIR2DL2 and co-stained for degranulation with CD107a. Results are from one representative experiment of three performed. Values represent means of triplicates. Bars, ±SD. Student’s unpaired *t*-test; **p < 0.01; ***p < 0.001; ns, not significant.

We consistently observed that GP expression by target cells reduced lysis by primary NK cells, although staining of HLA-I by W6/32 mAb was significantly reduced. We, therefore, assessed whether GP-mediated shielding of HLA-I from staining with the HLA-I-specific w6/32 mAb correlates with staining with a recombinant KIR2DL receptor that recognizes HLA-I. The HEK293T cell line (homozygous for HLA-C*07:01) was suitable for use in this assessment, since HEK293T cells express HLA-C allele that is recognized by the KIR2DL2 receptor (53, 54) but not the KIR2DL1 receptor. Our finding that high GP expression did not result in a reduction of staining by KIR2DL2-Ig (Figure 6A) indicated that GP did not mask the recognition of HLA by the KIR2DL2 receptor. We thus compared the levels of CD107a degranulation between KIR2DL2-positive and KIR2DL2-negative primary NK cells incubated with GP-transfected HEK293T target cells. Expression of the KIR2DL2 receptor by primary human NK cells reduced their degranulation response to both mock-transfected and GP-transfected target cells. However, the KIR2DL2-mediated reduction in degranulation was not affected by GP expression (Figure 6B, inset). These results were repeated in NK from additional donors and showed the same pattern (Figure S5C in Supplementary Material). Here, we should remember that, as shown above in Figure 5, expression of GP by target cells reduced NK cell function (Figure 6B). Note that incubation of NK with GP-transfected target cells did not affect the NK membrane-associated expression of NCRs, NKG2D, and KIR2DL2 as compared to NK cells incubated with mock-transfected cells (Figure S6 in Supplementary Material).

**Figure 7:**
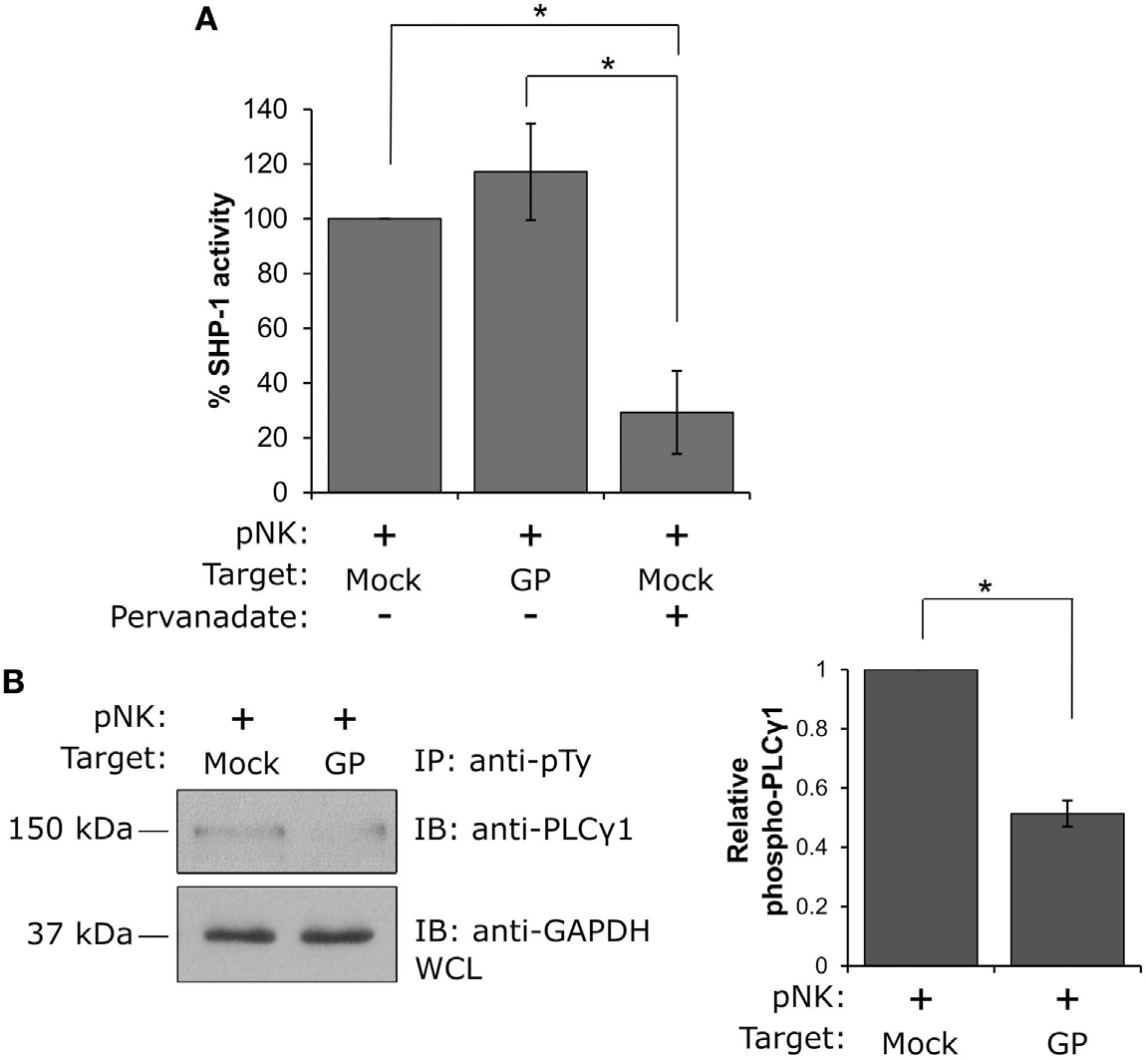
Natural killer (NK) cell interaction with GP-expressing cells suppresses NK cell activity by preventing PLCγ phosphorylation and not by promoting SHP-1 activity. **(A)** Primary NK cells were incubated with mock- or GP-transfected HEK293T target cells at 37°C for 5 min, and SHP-1 activity was determined as described in the Section “Materials and Methods.” NK cell lysis in the presence of the phosphatase inhibitor pervanadate served as a negative control. A bar graph summarizing SHP-1 catalytic activity obtained from three independent experiments is shown. **(B)** Primary NK cells were incubated with mock- or GP-transfected HEK293T target cells at 37°C for 5 min before lysis. Immunoprecipitation (IP) of phosphotyrosine immunoblotted using anti-PLCγ1 is shown. Since IP using anti-pTy antibody lacks a precipitation control, as a loading control, whole cell lysates (WCL) were analyzed by Western blotting with anti-GAPDH antibody. PLCγ1 band intensities from three independent experiments were determined by densitometric analysis and expressed relative to the band intensity of GAPDH. **(A,B)** Data in the bar graphs are means ± SEM of three independent experiments. Student’s unpaired *t*-test; *p < 0.05.

Suppression of NK cell activity by the GP protein can be attributed to one of two main mechanisms: (i) perturbation of signal transduction through activating receptors or (ii) triggering of inhibitory signals following engagement of KIR inhibitory receptors. To elucidate the mechanism of suppression of NK cell activity, we exploited previous findings showing that the activity of the SH2-domain-containing protein tyrosine phosphatase-1 (SHP-1) serves as the main inhibitory factor attenuating NK cell activation following inhibitory receptor engagement. This engagement leads to the recruitment and activation of SHP-1 at the natural killer immunology synapse, where it dephosphorylates key signaling molecules, including VAV1, LAT, and phospholipase Cγ1/2 (PLCγ1/2), thereby inhibiting NK cell activation (55, 56). We thus performed a phosphatase assay, in which the role of GP in controlling the catalytic activity of SHP-1 was examined. Primary human NK cells were incubated with HEK293T target cells expressing GP versus mock transfected cells, and the activity of SHP-1 was measured. Importantly, it was found that the interaction of NK cells with GP expressing target cells did not affect SHP-1 activity, relative to the mock-transfected cells (GP: 117.1 ± 17.7% versus mock: 100%, *p* = 0.4; Figure 7A). As a negative control, lysates of NK:Target conjugates were treated with the phosphatase inhibitor pervanadate, which blocks the SHP-1 catalytic domain. A significant reduction in SHP-1 activity was measured following pervanadate treatment, confirming the validity of the assay for this cellular system (mock-pervanadate: 29.2 ± 15.1% versus mock-untreated: 100%, *p* = 0.04 or GP untreated: 117.1 ± 17.7%, *p* = 0.02; Figure 7A). These results indicate that GP does not significantly increase SHP-1 activity, suggesting that an alternative mechanism is indeed responsible for the compromised activation of NK cells following interaction with GP-expressing target cells. Potentially, this mechanism involves the masking of some NK-activating receptors, preventing the transduction of activating signals through these specific receptors.

To further explore this mechanism, we examined the tyrosine phosphorylation status of PLCγ1, a cytoplasmic enzyme responsible for the intracellular Ca^2+^ flux that is essential for NK cell activation and function. Following the engagement of activating receptors, PLCγ1 phosphorylation is crucial for activation of NK cells. Primary NK cells were thus incubated with mock- or GP-transfected HEK293T cells, and IP experiments using anti-phosphotyrosine antibody were performed; immunoblotting with anti-PLCγ1 antibody revealed that while PLCγ1 was highly phosphorylated following NK cell interaction with mock transfected cells, interaction with GP-expressing cells resulted in a substantial reduction in PLCγ1 phosphorylation (Figure 7B). These data support the findings obtained by the NK functional assays (Figures 5 and 6) that suppression of NK cell activity following exposure to the GP protein may be attributed to the prevention of activating signal transduction and does not take place *via* activation of inhibitory signaling. Our data implies a mechanism in which GP could mask ligands to some NK-activating receptors on target cells, thereby preventing their engagement with NK cells.

## Discussion

Viral evasion of the immune system often involves modulation of host functions. In particular, interference with the expression of ligands for immune cell receptors is a ubiquitous—and well-studied—evasive strategy in different virus families (20–26). HLA-I, in particular, is a ligand that constitutes a major target in viral evasion. Nonetheless, interference with HLA-I is not always a beneficial tactic due to the opposing roles it plays in T cell versus NK cell activation. Downregulation of HLA-I exposes target cells to NK cell activity, while upregulation may increase host cell vulnerability to attack by cytotoxic T lymphocytes (CTLs). Therefore, HLA-I-based viral evasion mechanisms are usually coupled to a complementary element that addresses the consequent activation of either CTLs or NK cells. Among the many well-known examples of viruses that regulate HLA-I are cytomegaloviruses and flaviviruses. In the former, CTL evasion by the virus is mediated by downregulation of HLA-I (20–26) in a mechanism coupled to HLA-I mimicry by the viral UL18, which suppresses NK cells (57). Flaviviruses upregulate the expression of HLA-I, which hinders NK activity, leading to acute viremia in the early stages of infection. This induced high viremia, due to high HLA-I expression by infected cells, creates the conditions necessary for the effective infection by the virus of its arthropod vector. Therefore, the clear benefit to virus, in terms of transmission to its vector, justifies the price paid for allowing an effective adaptive response that clears the infection in the host after transmission to the vector (27). In contrast, another common but more straight-forward evasion mechanism is the downmodulation of activating stress-induced ligands, such as NKG2D ligands (19, 58, 59). Secretion of receptor blocking ligands, such as pp65 (60) and soluble ULBPs (61), constitutes yet another viral evasion strategy.

Our results did not show any direct interaction between purified SUDV-GP and various NK cell receptors; thus, we investigated the effect of membrane-associated expression of EBOV-GP on the interaction of GP-expressing cells and NK cells. HLA-I shielding has previously been reported for the GPs of the Zaire and Reston strains of EBOV (8, 9), and here we showed this phenomenon for the SUDV- and Makona-GPs. A significant steric shielding of HLA-I was observed only in high expression of GP [(9) and our results]. Alazard-Dany et al. (49) show that infection with recombinant EBOV results in loss of HLA-I staining only in late infection stage (48 h), whereas synthesis and massive release of virus particles occur at early infection steps. Yet, this late-stage shielding of ligands to immune receptors should reduce subsequent activation and development of anti-Ebola immunity that could cope with parallel infected cells that are in early infection stage.

Since HLA-I molecules serve mostly as ligands of NK inhibitory receptors, it was possible that the shielding mechanism would promote increased NK cell activity against GP-expressing cells. Yet, we observed that primary human NK cells were less reactive to GP-expressing HEK293T cells. We, therefore, studied the recognition of GP-expressing cells by NK-activating receptors and demonstrated that GP-expression in cells was manifested as a decrease in detection by recombinant NKG2D and NKp30 receptors. Similarly to HLA-I, this decrease was due to shielding, as enzymatic removal of cell-surface GP restored the recognition of ligands of the NKG2D and NKp30 receptors. This shielding of target-expressed ligands of NK activation receptors sufficed to reduce NK cell activity. Importantly, the shielding of target HLA-I, as observed by anti-HLA-I staining, did not inhibit the function of NK inhibitory receptors. This finding was in accordance with the observation that HLA-I-recognizing recombinant KIR did not reduce binding to GP-expressing cells as compared to recombinant NKG2D and NKp30 receptors. We may, therefore, conclude that GP partially masks the HLA molecules: it leaves them exposed to KIR binding while blocking the TCRs and the W6/32 antibody (that we employed in this study). Indeed, it has been found that TCRs and KIRs have different modes of binding to HLA-I (62). HLA-I-based selective shielding to TCRs but not to KIRs and shielding of ligands to NK activation receptors is in accordance with reports of reduced T and NK cell function in EBOV patients (16, 17, 50, 62).

Natural killer cells are innate lymphocytes that are known to mount a strong response against virus-infected cells (11). However, studies of EBOV infection in primates showed a decrease in the activity and numbers of NK cells, a surprising finding, since HLA-I is an inhibitory ligand for NK cell receptors, such as KIRs (15–17). In light of these studies, we investigated the effect of membrane-associated expression of SUDV-GP on (i) the interaction of NK cell activation and inhibitory receptors with their ligands, and (ii) its effect on the function of human primary NK cells. We found that while NKG2D and NKp30 fusion-Ig recognition of GP-expressing HEK293T cells was blocked by SUDV-GP, the inhibitory receptor KIR2DL2 was not blocked and the inhibitory signal from KIR2DL2 was not reduced. This result can explain the low activity of NK cells in EBOV infection in primate models (32, 63).

The observed functionality of NK-expressed KIR2DL2 on the GP-expressing cells could point to a differential shielding of HLA-A and B versus HLA-C by GP. A similar discrimination between HLA-A and B versus HLA-C was reported for the HIV-NEF protein that interact with HLA-A and B through a tyrosine residue missing absent in the HLA-C. This selective HIV-NEF-mediated downregulation of HLA-A and B, but not HLA-C, was reported to protect HIV-infected cells from NK cells (64). Interestingly, an activating KIR profile was associated with fatal outcome of Ebola infection and this deleterious effect seems linked to KIR2DS1 and KIR2DS3 genes that were by far more present among non-survivors (65). If indeed Ebola-GP does not shield membrane-associated HLA-C, then it will not interfere with the HLA-C-KIR2DS1 interaction (HLA-C2 allele is the target for KIR2DS1). This could corroborate with findings that Ebola-mediated over-activation of the immune response is responsible for the rapid depletion in NK cells (65) and also fit the aforementioned observations that EBOV infection in primates showed a decrease in the activity and numbers of NK cells (15–17). The seeming contradiction between shielding ligands to NKG2D and NKp30 while sparing ligands to KIR-based activation and inhibitory receptors indicate the limitations of this current study that is not focusing on the NK receptor profile of the host, which could better explain the NK response to natural EBOLA infection.

The above discussion leads naturally to the VSVΔG/EBOV GP vaccine, a recent development in the field of filoviruses, as this vaccine offers substantial protection against EBOV disease (66, 67). Fourteen days after vaccination, macaques developed neutralizing IgG antibodies against EBOV-GP (63). Moreover, the vaccinated macaques exhibited increased levels of IFNα, IL-15, and IFNγ, 3 days post EBOV challenge. The significance of the elevated levels of IL-15, which is secreted by activated macrophages in EBOV infection, lies in the fact that IL-15 controls NK cell numbers. Furthermore, NK cells can inflict antibody-dependent cell cytotoxicity (ADCC), which may contribute greatly to the effect of antibodies directed against viral proteins (68, 69). Upon treatment with several anti EBOV mAbs, NK cell-deficient mice had lower viral clearance as compared to WT mice, when infected with an EBOV-glycoprotein pseudotyped human immunodeficiency virus, indicating that the anti-EBOV mechanism of the ADCC activity of these mAbs is predominantly mediated by NK cells (70). Thus, it appears that the VSVDG/EBOV GP vaccine may act to neutralize EBOV viral particles, but it may also promote the activation of NK cells *via* ADCC This effect of the VSVΔG/EBOV GP vaccine may shift the inhibition–activation balance of NK cells toward activation, as is indicated by our results, thereby overcoming the EBOV shielding of ligands to NK cells activating receptors, as in the case of NKG2D and NKp30 ligands, and allowing the elimination of EBOV-infected cells.

In summary, we showed that activating and inhibitory ligands for NK receptors are differentially shielded by Ebola GP membrane-associated expression. These findings indicate a novel evasion mechanism employed by the EBOV to specifically avoid the NK cell immune response.

## Author Contributions

AE, LL, and AP initiated the project; AE, AS, OM, MB-S, MI, UH and AP planned, performed, and analyzed experiments; AE, MB, OR, OG-Y, JD, OMan, LL, and AP designed and prepared required cell cultures, expression vectors, and reagents. JD, OMan, and LL reviewed the manuscript. AE, AS, and AP wrote the manuscript.

## Conflict of Interest Statement

The authors declare that the research was conducted in the absence of any commercial or financial relationships that could be construed as a potential conflict of interest.

## Supplementary Material

Figure S1 Low GP expressers and Mock-transfected cells are stained similarly for cell-surface human leukocyte antigen class-1 (HLA-I) molecules and NCR ligands. Mock transfection of cells was performed exactly as GP transfection was performed, yet with the pCAGGS plasmid backbone instead of the GP-pCAGGS vector. Mock-transfected cells were harvested and stained for HLA-I and MICA as described above or stained with NKG2D-Ig and NKp30-Ig as described above. Histograms show the overlay of mock-transfected cells with low-GP expressers from the same experiments. Results shown are from one representative analysis of 30 independent flow cytometry analyses.

Figure S2 Intracellular levels of human leukocyte antigen class-1 (HLA-I) and MICA are not affected from GP transfection. HEK293T cells were transfected, harvested, and stained for HLA-I and MICA cell-surface expression as described before **(A,C)** or stained in the presence of the true-nuclear transcription factor buffer set, which permeabilized and fixed the cells to ensure intracellular staining **(B,D)**. **(E)** HEK293T cells were co-transfected with MICA-green fluorescent protein and GP-YFP and analyzed without further staining or permeabilization in the flow cytometer. **(F–I)** H5-transfected HEK293T cells were harvested and stained with allophycocyanin-conjugated anti-H5 together with staining with NKG2D-Ig/NKp30-Ig/NKp44-Ig/hFc as described before. Results are from one representative experiment of two performed.

Figure S3 Surface GP expression is sensitive to trypsin treatment, while HLA-I, MICA, and B7-H6 are only partly affected by the same trypsin treatment protocol. **(A)** Representative flow cytometry analysis for the effect of a short exposure to trypsin on the expression of membrane-associated molecules. HEK293T cells were harvested, incubated in the presence of trypsin for either 2.5 or 5 min or left untreated, and stained for HLA-A, B, C, MICA, or B7-H6 surface antigens with phycoerythrin (PE)-conjugated antibodies. Alternatively, cells were transfected with Sudan virus (SUDV)-GP, harvested, incubated in the presence of trypsin for either 2.5 or 5 min, or left untreated and stained for SUDV-GP using biotinylated 3C10 antibody, followed by allophycocyanin-conjugated streptavidin. Dead cells were excluded using 7-aminoactinomycin D. **(B)** HEK293T cells were transfected with SUDV-GP, harvested, treated with DTT as previously described (9), and stained for HLA-A, B, C, or MICA surface antigens with PE-conjugated antibodies. **(C)** HEK293T cells were harvested, incubated in the presence of trypsin for 2.5 min, washed, and re-placed in 37c in aliquots. Cells were stained for both GP and HLA-I expression as before in different time points following trypsin digestion. Percent GP expression represent percent GP positive cells as compared to trypsin untreated cells; recovered cells represented same GP staining pattern as trypsin non-treated cells. Percent shielding level represent the fraction of HLA-I negative cells as compared to the fraction of the HLA-I negative cells in the trypsin non-treated cells. Results are from one representative experiment of three [**(A)** trypsin time titration] and two **(B,C)** performed.

Figure S4 Gating strategies applied in FACS functional assays. Effector and target cells were prepared as previously described, stained, and analyzed using the following sequences: **(A)** degranulation assay analysis (71): single cells were gated as depicted in scheme on a FSC-H/FSC-A plot. Live pNK cells were then further gated on a SSC-A/FSC-A plot followed by gating on a 7-aminoactinomycin D (7AAD) histogram. To exclude remaining target cells, CD16-positive cells were gated and plotted on KIR2DL2/CD107a plot. **(B)** Specific lysis assay analysis (43): target cell population was gated on carboxyfluorescein succinimidyl ester/FSC-A plot, debris and apoptotic bodies were excluded on a 7AAD/FSC-A plot, GP+ and GP− cells were segregated by gating on a GP-allophycocyanin histogram and plotted on 7AAD/FSC-A plot to determine population specific live/dead ratio.

Figure S5 Glycoprotein-mediated downmodulation of pNK activation from different donors. **(A)** CD107a FACS-based degranulation assay was performed as previously described, results from four different donors are depicted. **(B)** IFNγ ELISA-based cytokine secretion assay was performed as previously described, results from four different donors are depicted. Results are from one representative experiment of two performed. **(C)** CD107a FACS-based degranulation assay, including KIRR2DL2 staining, was performed as previously described, results from four different donors are depicted. Values represent means of triplicates. Bars, ±SD.

Figure S6 Co incubation of pNK cell with GP expressing cell does not affect NCR expression nor the expression of NKG2D and KIR2DL2. HEK293T cells were either SUDV-GP transfected or mock transfected and cocultured with pNK cells in the presence of 25 U/ml rhIL2. Cells were then harvested and pNK cells were analyzed for NKr expression by flow cytometry. Dead cells were excluded by 7-aminoactinomycin D; pNK cells were then gated by staining for CD16 and co-stained for either NKp30, NKp44, NKp46, NKG2D, or KIR2DL2.

## References

[1] Leroy E Gonzalez JP Pourrut X. Ebolavirus and other filoviruses. Curr Top Microbiol Immunol (2007) 315:363–87. 10.1007/978-3-540-70962-6_15 17848072 PMC7121322

[2] Lee JE Saphire EO. Ebolavirus glycoprotein structure and mechanism of entry. Future Virol (2009) 4(6):621–35. 10.2217/fvl.09.56 20198110 PMC2829775

[3] Ray RB Basu A Steele R Beyene A McHowat J Meyer K Ebola virus glycoprotein-mediated anoikis of primary human cardiac microvascular endothelial cells. Virology (2004) 321(2):181–8. 10.1016/j.virol.2003.12.014 15051379

[4] Simmons G Wool-Lewis RJ Baribaud F Netter RC Bates P. Ebola virus glycoproteins induce global surface protein down-modulation and loss of cell adherence. J Virol (2002) 76(5):2518–28. 10.1128/jvi.76.5.2518-2528.2002 11836430 PMC153797

[5] Yang ZY Duckers HJ Sullivan NJ Sanchez A Nabel EG Nabel GJ. Identification of the Ebola virus glycoprotein as the main viral determinant of vascular cell cytotoxicity and injury. Nat Med (2000) 6(8):886–9. 10.1038/78645 10932225

[6] Takada A Watanabe S Ito H Okazaki K Kida H Kawaoka Y. Downregulation of beta1 integrins by Ebola virus glycoprotein: implication for virus entry. Virology (2000) 278(1):20–6. 10.1006/viro.2000.0601 11112476

[7] Chan SY Ma MC Goldsmith MA. Differential induction of cellular detachment by envelope glycoproteins of Marburg and Ebola (Zaire) viruses. J Gen Virol (2000) 81(9):2155–9. 10.1099/0022-1317-81-9-2155 10950971

[8] Noyori O Matsuno K Kajihara M Nakayama E Igarashi M Kuroda M Differential potential for envelope glycoprotein-mediated steric shielding of host cell surface proteins among filoviruses. Virology (2013) 446(1–2):152–61. 10.1016/j.virol.2013.07.029 24074577

[9] Francica JR Varela-Rohena A Medvec A Plesa G Riley JL Bates P. Steric shielding of surface epitopes and impaired immune recognition induced by the Ebola virus glycoprotein. PLoS Pathog (2010) 6(9):e1001098. 10.1371/journal.ppat.1001098 20844579 PMC2936550

[10] Kärre K Ljunggren HG Piontek G Kiessling R Selective rejection of H-2-deficient lymphoma variants suggests alternative immune defence strategy. Nature (1986) 319(6055):675–8. 10.1038/319675a0 3951539

[11] Vivier E Raulet DH Moretta A Caligiuri MA Zitvogel L Lanier LL Innate or adaptive immunity? The example of natural killer cells. Science (2011) 331(6013):44–9. 10.1126/science.1198687 21212348 PMC3089969

[12] Bauer S Groh V Wu J Steinle A Phillips JH Lanier LL Activation of NK cells and T cells by NKG2D, a receptor for stress-inducible MICA. Science (1999) 285(5428):727–9. 10.1126/science.285.5428.727 10426993

[13] Liu X Speranza E Muñoz-Fontela C Haldenby S Rickett NY Garcia-Dorival I Transcriptomic signatures differentiate survival from fatal outcomes in humans infected with Ebola virus. Genome Biol (2017) 18(1):4. 10.1186/s13059-016-1137-3 28100256 PMC5244546

[14] Cimini E Viola D Cabeza-Cabrerizo M Romanelli A Tumino N Sacchi A Different features of Vδ2 T and NK cells in fatal and non-fatal human Ebola infections. PLoS Negl Trop Dis (2017) 11(5):e0005645. 10.1371/journal.pntd.0005645 28558022 PMC5472323

[15] Ignatiev GM Dadaeva AA Luchko SV Chepurnov AA. Immune and pathophysiological processes in baboons experimentally infected with Ebola virus adapted to guinea pigs. Immunol Lett (2000) 71(2):131–40. 10.1016/S0165-2478(99)00169-8 10714441

[16] Reed DS Hensley LE Geisbert JB Jahrling PB Geisbert TW. Depletion of peripheral blood T lymphocytes and NK cells during the course of ebola hemorrhagic fever in cynomolgus macaques. Viral Immunol (2004) 17(3):390–400. 10.1089/vim.2004.17.390 15357905

[17] Geisbert TW Hensley LE Larsen T Young HA Reed DS Geisbert JB Pathogenesis of Ebola hemorrhagic fever in cynomolgus macaques. Am J Pathol (2003) 163(6):2347–70. 10.1016/S0002-9440(10)63591-2 14633608 PMC1892369

[18] Williams KJN Qiu X Fernando L Jones SM Alimonti JB. VSVΔG/EBOV GP-induced innate protection enhances natural killer cell activity to increase survival in a lethal mouse adapted Ebola virus infection. Viral Immunol (2015) 28(1):51–61. 10.1089/vim.2014.0069 25494457

[19] Seidel E Le VTK Bar-On Y Tsukerman P Enk J Yamin R Dynamic co-evolution of host and pathogen: HCMV downregulates the prevalent allele MICA*008 to escape elimination by NK cells. Cell Rep (2015) 10(6):968–82. 10.1016/j.celrep.2015.01.029 PMC4641326 25683719

[20] Hengel H Koopmann JO Flohr T Muranyi W Goulmy E Hämmerling GJ A viral ER-resident glycoprotein inactivates the MHC-endoced peptide transporter. Immunity (1997) 6(5):623–32. 10.1016/S1074-7613(00)80350-7 9175840

[21] Ahn K Gruhler A Galocha B Jones TR Wiertz EJHJ Ploegh HL The ER-luminal domain of the HCMV glypcoprotein US6 inhibits peptide translocation by TAP. Immunity (1997) 6(5):613–21. 10.1016/S1074-7613(00)80349-0 9175839

[22] Gruhler A Peterson PA Früh K. Human cytomegalovirus immediate early glycoprotein US3 retains MHC class I molecules by transient association. Traffic (2000) 1(4):318–25. 10.1034/j.1600-0854.2000.010405.x 11208117

[23] Jones TR Wiertz EJ Sun L Fish KN Nelson JA Ploegh HL. Human cytomegalovirus US3 impairs transport and maturation of major histocompatibility complex class I heavy chains. Proc Natl Acad Sci U S A (1996) 93(21):11327–33. 10.1073/pnas.93.21.11327 8876135 PMC38057

[24] Gewurz BE Wang EW Tortorella D Schust DJ Ploegh HL. Human cytomegalovirus US2 endoplasmic reticulum-lumenal domain dictates association with major histocompatibility complex class I in a locus-specific manner. J Virol (2001) 75(11):5197–204. 10.1128/JVI.75.11.5197-5204.2001 11333901 PMC114925

[25] Wiertz EJ Tortorella D Bogyo M Yu J Mothes W Jones TR Sec61-mediated transfer of a membrane protein from the endoplasmic reticulum to the proteasome for destruction. Nature (1996) 384:432–8. 10.1038/384432a0 8945469

[26] Wiertz EJHJ Jones TR Sun L Bogyo M Geuze HJ Ploegh HL. The human cytomegalovirus US11 gene product dislocates MHC class I heavy chains from the endoplasmic reticulum to the cytosol. Cell (1996) 84(5):769–79. 10.1016/S0092-8674(00)81054-5 8625414

[27] Hershkovitz O Zilka A Bar-Ilan A Abutbul S Davidson A Mazzon M Dengue virus replicon expressing the nonstructural proteins suffices to enhance membrane expression of HLA class I and inhibit lysis by human NK cells. J Virol (2008) 82(15):7666–76. 10.1128/JVI.02274-07 18508882 PMC2493327

[28] Wilkinson GWG Tomasec P Stanton RJ Armstrong M Prod’homme V Aicheler R Modulation of natural killer cells by human cytomegalovirus. J Clin Virol (2008) 41(3):206–12. 10.1016/j.jcv.2007.10.027 18069056 PMC2843162

[29] Stewart CA Laugier-Anfossi F Vély F Saulquin X Riedmuller J Tisserant A Recognition of peptide-MHC class I complexes by activating killer immunoglobulin-like receptors. Proc Natl Acad Sci U S A (2005) 102(37):13224–9. 10.1073/pnas.0503594102 16141329 PMC1201584

[30] Mahanty S Gupta M Paragas J Bray M Ahmed R Rollin PE. Protection from lethal infection is determined by innate immune responses in a mouse model of Ebola virus infection. Virology (2003) 312(2):415–24. 10.1016/S0042-6822(03)00233-2 12919746

[31] Bray M Geisbert TW. Ebola virus: the role of macrophages and dendritic cells in the pathogenesis of Ebola hemorrhagic fever. Int J Biochem Cell Biol (2005) 37(8):1560–6. 10.1016/j.biocel.2005.02.018 15896665

[32] Warfield KL Perkins JG Swenson DL Deal EM Bosio CM Aman MJ Role of natural killer cells in innate protection against lethal Ebola virus infection. J Exp Med (2004) 200(2):169–79. 10.1084/jem.20032141 15249592 PMC2212007

[33] Gruda R Brown ACN Grabovsky V Mizrahi S Gur C Feigelson SW Loss of kindlin-3 alters the threshold for NK cell activation in human leukocyte adhesion deficiency-III. Blood (2012) 120(19):3915–24. 10.1182/blood-2012-02-410795 22983444

[34] Misasi J Chandran K Yang J-Y Considine B Filone CM Côté M Filoviruses require endosomal cysteine proteases for entry but exhibit distinct protease preferences. J Virol (2012) 86(6):3284–92. 10.1128/JVI.06346-11 22238307 PMC3302294

[35] Gire SK Goba A Andersen KG Sealfon RSG Park DJ Kanneh L Genomic surveillance elucidates Ebola virus origin and transmission during the 2014 outbreak. Science (2014) 345(6202):1369–72. 10.1126/science.1259657 25214632 PMC4431643

[36] Baize S Pannetier D Oestereich L Rieger T Koivogui L Magassouba N Emergence of Zaire Ebola virus disease in Guinea. N Engl J Med (2014) 371(15):1418–25. 10.1056/NEJMoa1404505 24738640

[37] Stern-Ginossar N Gur C Biton M Horwitz E Elboim M Stanietsky N Human microRNAs regulate stress-induced immune responses mediated by the receptor NKG2D. Nat Immunol (2008) 9(9):1065–73. 10.1038/ni.1642 18677316

[38] Bloushtain N Qimron U Bar-Ilan A Hershkovitz O Gazit R Fima E Membrane-associated heparan sulfate proteoglycans are involved in the recognition of cellular targets by NKp30 and NKp46. J Immunol (2004) 173(4):2392–401. 10.4049/jimmunol.173.4.2392 15294952

[39] Rosental B Brusilovsky M Hadad U Oz D Appel MY Afergan F Proliferating cell nuclear antigen is a novel inhibitory ligand for the natural cytotoxicity receptor NKp44. J Immunol (2011) 187(11):5693–702. 10.4049/jimmunol.1102267 22021614 PMC3269963

[40] Mandelboim O Lieberman N Lev M Paul L Arnon TI Bushkin Y Recognition of haemagglutinins on virus-infected cells by NKp46 activates lysis by human NK cells. Nature (2001) 409(6823):1055–60. 10.1038/35059110 11234016

[41] Brusilovsky M Cordoba M Rosental B Hershkovitz O Andrake MD Pecherskaya A Genome-wide siRNA screen reveals a new cellular partner of NK cell receptor KIR2DL4: heparan sulfate directly modulates KIR2DL4-mediated responses. J Immunol (2013) 191:5256–67. 10.4049/jimmunol.1302079 24127555 PMC3836631

[42] Gonen-Gross T Goldman-Wohl D Huppertz B Lankry D Greenfield C Natanson-Yaron S Inhibitory NK receptor recognition of HLA-G: regulation by contact residues and by cell specific expression at the fetal-maternal interface. PLoS One (2010) 5(1):e8941. 10.1371/journal.pone.0008941 20126612 PMC2812487

[43] Lecoeur H Février M Garcia S Rivière Y Gougeon ML. A novel flow cytometric assay for quantitation and multiparametric characterization of cell-mediated cytotoxicity. J Immunol Methods (2001) 253(1–2):177–87. 10.1016/S0022-1759(01)00359-3 11384679

[44] Lorenz U Protein tyrosine phosphatase assays. Curr Protoc Immunol (2011) Chapter 11:Unit 11.7. 10.1002/0471142735.im1107s93 PMC3097125 21462163

[45] Hornung V Rothenfusser S Britsch S Krug A Jahrsdörfer B Giese T Quantitative expression of toll-like receptor 1-10 mRNA in cellular subsets of human peripheral blood mononuclear cells and sensitivity to CpG oligodeoxynucleotides. J Immunol (2002) 168(9):4531–7. 10.4049/jimmunol.168.9.4531 11970999

[46] Souza-Fonseca-Guimaraes F Parlato M Philippart F Misset B Cavaillon J-M Adib-Conquy M Toll-like receptors expression and interferon-gamma production by NK cells in human sepsis. Crit Care (2012) 16(5):R206 10.1186/cc11838 23098236 PMC3682310

[47] Lai CY Strange DP Wong TAS Lehrer AT Verma S. Ebola virus glycoprotein induces an innate immune response in vivo via TLR4. Front Microbiol (2017) 8(8):1571. 10.3389/fmicb.2017.01571 28861075 PMC5562721

[48] Okumura A Pitha PM Yoshimura A Harty RN. Interaction between Ebola virus glycoprotein and host toll-like receptor 4 leads to induction of proinflammatory cytokines and SOCS1. J Virol (2010) 84(1):27–33. 10.1128/JVI.01462-09 19846529 PMC2798428

[49] Alazard-Dany N Volchkova V Reynard O Carbonnelle C Dolnik O Ottmann M Ebola virus glycoprotein GP is not cytotoxic when expressed constitutively at a moderate level. J Gen Virol (2006) 87(5):1247–57. 10.1099/vir.0.81361-0 16603527

[50] Mahanty S Bray M Pathogenesis of filoviral haemorrhagic fevers. Lancet Infect Dis (2004) 4:487–98. 10.1016/S1473-3099(04)01103-X 15288821

[51] Martines RB Ng DL Greer PW Rollin PE Zaki SR. Tissue and cellular tropism, pathology and pathogenesis of Ebola and Marburg viruses. J Pathol (2015) 235(2):153–74. 10.1002/path.4456 25297522

[52] Wool-Lewis RJ Bates P. Endoproteolytic processing of the ebola virus envelope glycoprotein: cleavage is not required for function. J Virol (1999) 73(2):1419–26. 9882347 10.1128/jvi.73.2.1419-1426.1999 PMC103966

[53] Lanier LL. NK cell recognition. Annu Rev Immunol (2005) 23(1):225–74. 10.1146/annurev.immunol.23.021704.115526 15771571

[54] Parham P Moffett A. Variable NK cell receptors and their MHC class I ligands in immunity, reproduction and human evolution. Nat Rev Immunol (2013) 13(2):133–44. 10.1038/nri3370 23334245 PMC3956658

[55] Matalon O Fried S Ben-Shmuel A Pauker MH Joseph N Keizer D Dephosphorylation of the adaptor LAT and phospholipase C-γ by SHP-1 inhibits natural killer cell cytotoxicity. Sci Signal (2016) 9(429):ra54. 10.1126/scisignal.aad6182 27221712

[56] Stebbins CC Watzl C Billadeau DD Leibson PJ Burshtyn DN Long EO. Vav1 dephosphorylation by the tyrosine phosphatase SHP-1 as a mechanism for inhibition of cellular cytotoxicity. Mol Cell Biol (2003) 23(17):6291–9. 10.1128/MCB.23.17.6291-6299.2003 12917349 PMC180957

[57] Chapman TL Heikema AP Bjorkman PJ. The inhibitory receptor LIR-1 uses a common binding interaction to recognize class I MHC molecules and the viral homolog UL18. Immunity (1999) 11(5):603–13. 10.1016/S1074-7613(00)80135-1 10591185

[58] Welte SA Sinzger C Lutz SZ Singh-Jasuja H Sampaio KL Eknigk U Selective intracellular retention of virally induced NKG2D ligands by the human cytomegalovirus UL16 glycoprotein. Eur J Immunol (2003) 33:194–203. 10.1002/immu.200390022 12594848

[59] Dunn C Chalupny NJ Sutherland CL Dosch S Sivakumar PV Johnson DC Human cytomegalovirus glycoprotein UL16 causes intracellular sequestration of NKG2D ligands, protecting against natural killer cell cytotoxicity. J Exp Med (2003) 197(11):1427–39. 10.1084/jem.20022059 12782710 PMC2193902

[60] Wills MR Carmichael AJ Mynard K Jin X Weekes MP Plachter B The human cytotoxic T-lymphocyte (CTL) response to cytomegalovirus is dominated by structural protein pp65: frequency, specificity, and T-cell receptor usage of pp65-specific CTL. J Virol (1996) 70(11):7569–79. 8892876 10.1128/jvi.70.11.7569-7579.1996 PMC190825

[61] Song H Kim JK Cosman D Choi I. Soluble ULBP suppresses natural killer cell activity via down-regulating NKG2D expression. Cell Immunol (2006) 239(1):22–30. 10.1016/j.cellimm.2006.03.002 16630603

[62] Kuroki K Furukawa A Maenaka K. Molecular recognition of paired receptors in the immune system. Front Microbiol (2012) 3:429. 10.3389/fmicb.2012.00429 23293633 PMC3533184

[63] Marzi A Robertson SJ Haddock E Feldmann F Hanley PW Scott DP VSV-EBOV rapidly protects macaques against infection with the 2014/15 Ebola virus outbreak strain. Science (2015) 349(6249):739–42. 10.1126/science.aab3920 26249231 PMC11040598

[64] Cohen GB Gandhi RT Davis DM Mandelboim O Chen BK Strominger JL The selective downregulation of class I major histocompatibility complex proteins by HIV-1 protects HIV-infected cells from NK cells. Immunity (1999) 10(6):661–71. 10.1016/S1074-7613(00)80065-5 10403641

[65] Wauquier N Padilla C Becquart P Leroy E Vieillard V. Association of KIR2DS1 and KIR2DS3 with fatal outcome in Ebola virus infection. Immunogenetics (2010) 62(11–12):767–71. 10.1007/s00251-010-0480-x 20878400 PMC2978320

[66] Henao-Restrepo AM Camacho A Longini IM Watson CH Edmunds WJ Egger M Efficacy and effectiveness of an rVSV-vectored vaccine in preventing Ebola virus disease: final results from the Guinea ring vaccination, open-label, cluster-randomised trial (Ebola Ça Suffit!). Lancet (2017) 389(10068):505–18. 10.1016/S0140-6736(16)32621-6 28017403 PMC5364328

[67] Trad MA Naughton W Yeung A Mazlin L O’Sullivan M Gilroy N Ebola virus disease: an update on current prevention and management strategies. J Clin Virol (2017) 86:5–13. 10.1016/j.jcv.2016.11.005 27893999

[68] Biron CA Nguyen KB Pien GC Cousens LP Salazar-Mather TP. Natural killer cells in antiviral defense: function and regulation by innate cytokines. Annu Rev Immunol (1999) 17:189–220. 10.1146/annurev.immunol.17.1.189 10358757

[69] Vivier E Tomasello E Baratin M Walzer T Ugolini S. Functions of natural killer cells. Nat Immunol (2008) 9(5):503–10. 10.1038/ni1582 18425107

[70] Liu Q Fan C Li Q Zhou S Huang W Wang L Antibody-dependent-cellular-cytotoxicity-inducing antibodies significantly affect the post-exposure treatment of Ebola virus infection. Sci Rep (2017) 7:45552. 10.1038/srep45552 28358050 PMC5372081

[71] Bryceson YT Fauriat C Nunes JM Wood SM Björkström NK Long EO Functional analysis of human NK cells by flow cytometry. Methods Mol Biol (2010) 612:335–52. 10.1007/978-1-60761-362-6_23 20033652 PMC4969010

