# Supplementary figures and images for: The Ebola-Glycoprotein Modulates the Function of Natural Killer Cells
Source: Front Immunol. 2018 Jul 2;9:1428. doi: 10.3389/fimmu.2018.01428 (PMC6036185; doi:10.3389/fimmu.2018.01428)

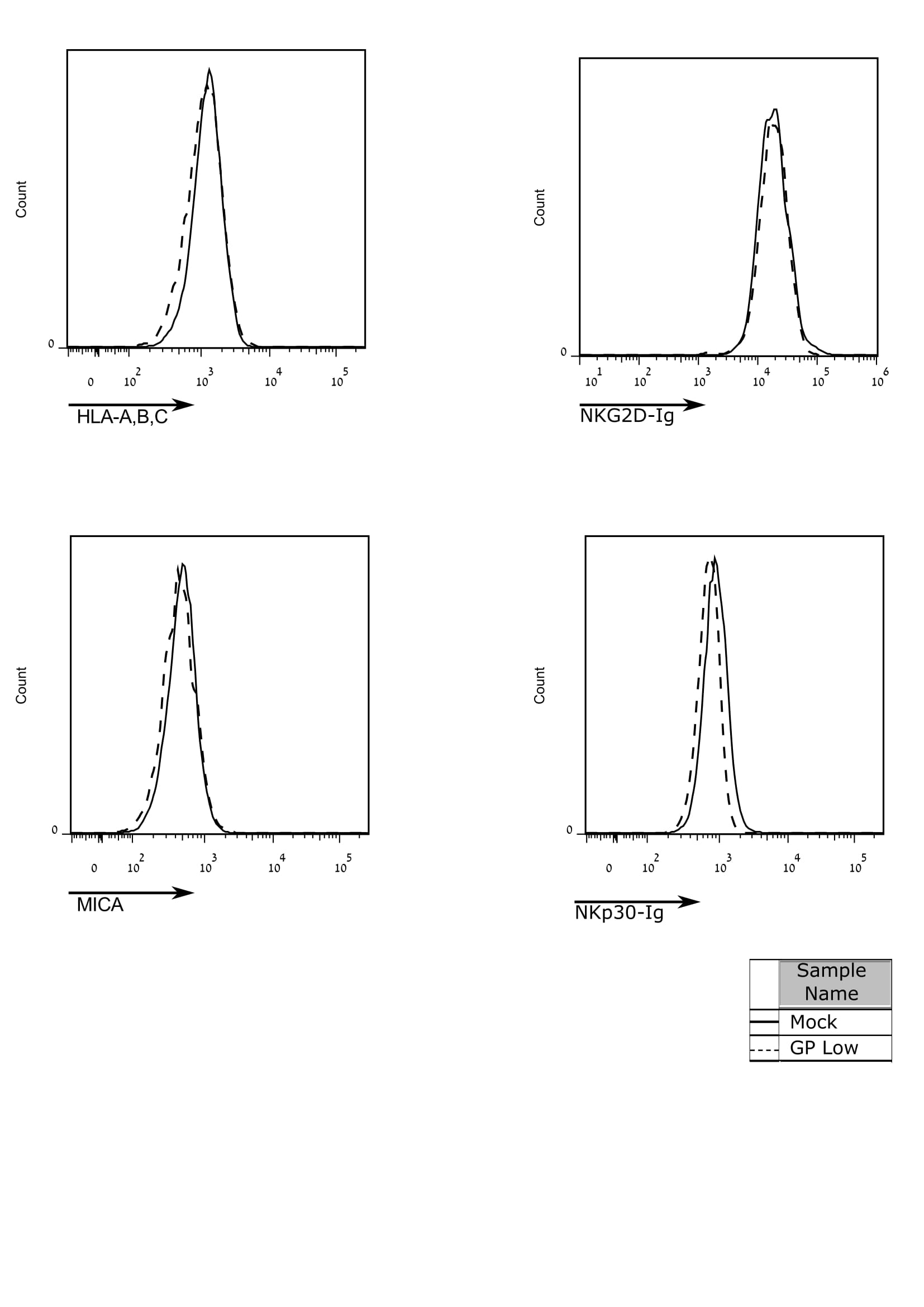

Supplement: Figure S1 — Low GP expressers and Mock-transfected cells are stained similarly for cell-surface human leukocyte antigen class-1 (HLA-I) molecules and NCR ligands. Mock transfection of cells was performed exactly as GP transfection was performed, yet with the pCAGGS plasmid backbone instead of the GP-pCAGGS vector. Mock-transfected cells were harvested and stained for HLA-I and MICA as described above or stained with NKG2D-Ig and NKp30-Ig as described above. Histograms show the overlay of mock-transfected cells with low-GP expressers from the same experiments. Results shown are from one representative analysis of 30 independent flow cytometry analyses. [file image_1.JPEG]

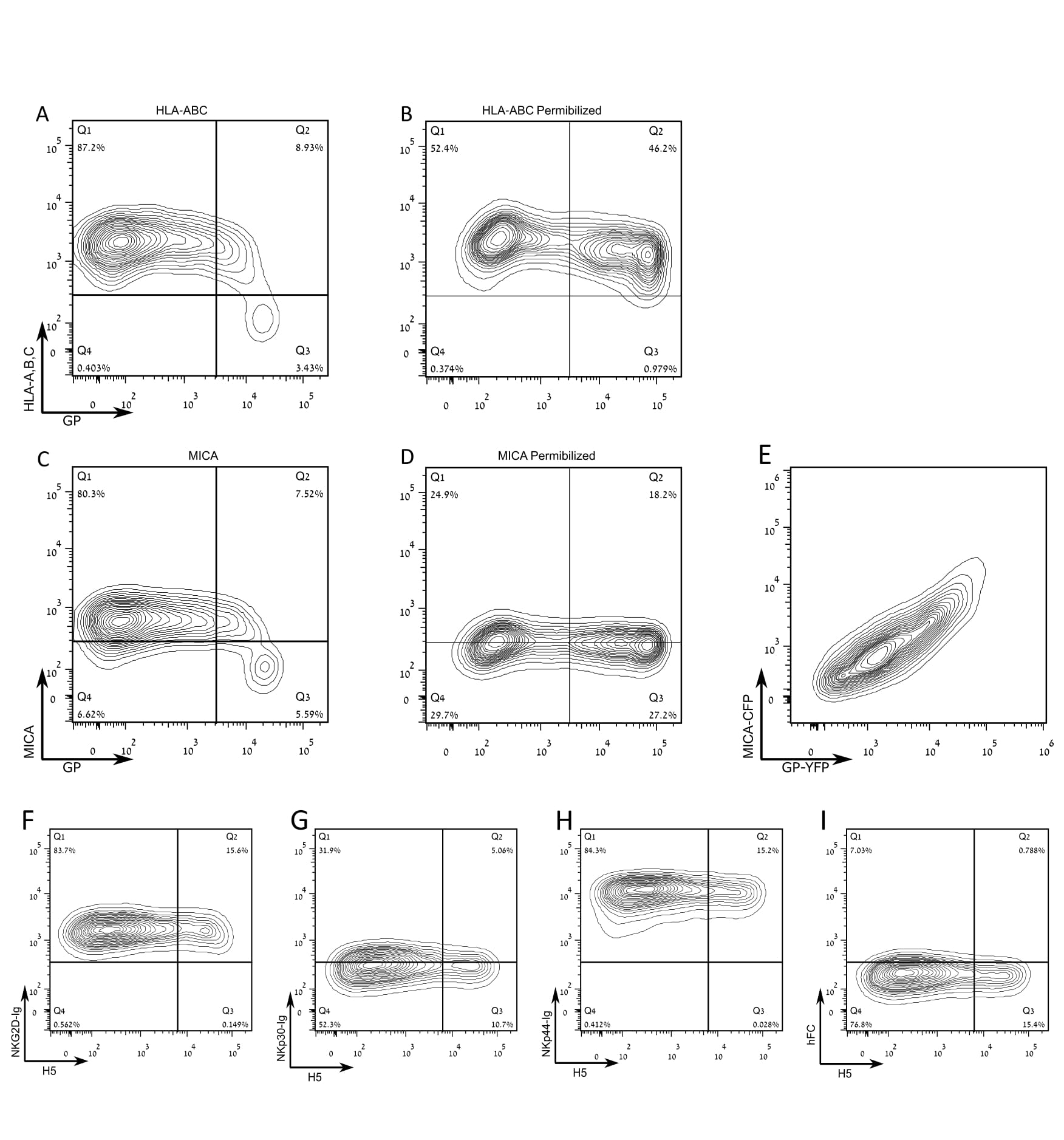

Supplement: Figure S2 — Intracellular levels of human leukocyte antigen class-1 (HLA-I) and MICA are not affected from GP transfection. HEK293T cells were transfected, harvested, and stained for HLA-I and MICA cell-surface expression as described before (A,C) or stained in the presence of the true-nuclear transcription factor buffer set, which permeabilized and fixed the cells to ensure intracellular staining (B,D). (E) HEK293T cells were co-transfected with MICA-green fluorescent protein and GP-YFP and analyzed without further staining or permeabilization in the flow cytometer. (F–I) H5-transfected HEK293T cells were harvested and stained with allophycocyanin-conjugated anti-H5 together with staining with NKG2D-Ig/NKp30-Ig/NKp44-Ig/hFc as described before. Results are from one representative experiment of two performed. [file image_2.JPEG]

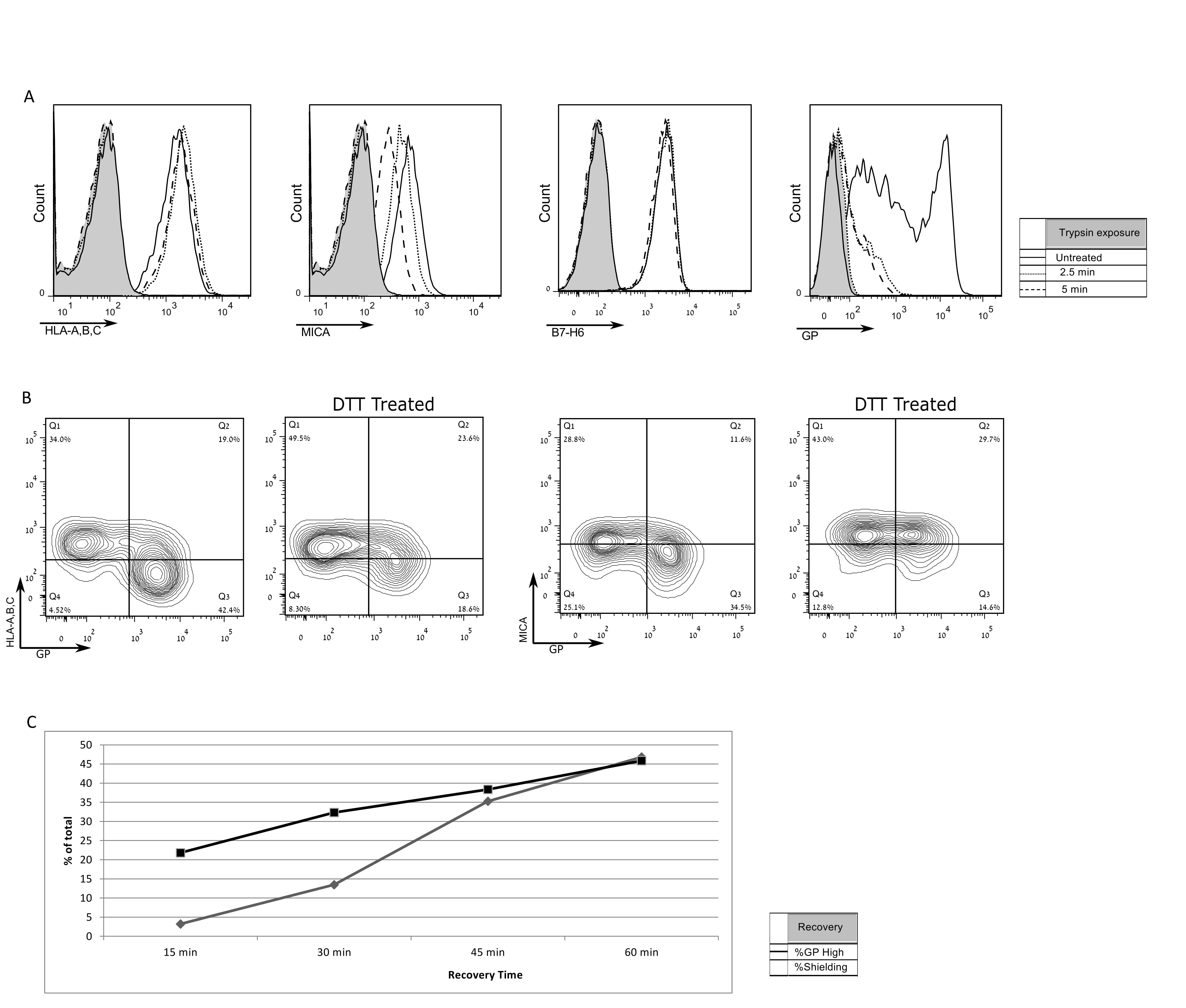

Supplement: Figure S3 — Surface GP expression is sensitive to trypsin treatment, while HLA-I, MICA, and B7-H6 are only partly affected by the same trypsin treatment protocol. (A) Representative flow cytometry analysis for the effect of a short exposure to trypsin on the expression of membrane-associated molecules. HEK293T cells were harvested, incubated in the presence of trypsin for either 2.5 or 5 min or left untreated, and stained for HLA-A, B, C, MICA, or B7-H6 surface antigens with phycoerythrin (PE)-conjugated antibodies. Alternatively, cells were transfected with Sudan virus (SUDV)-GP, harvested, incubated in the presence of trypsin for either 2.5 or 5 min, or left untreated and stained for SUDV-GP using biotinylated 3C10 antibody, followed by allophycocyanin-conjugated streptavidin. Dead cells were excluded using 7-aminoactinomycin D. (B) HEK293T cells were transfected with SUDV-GP, harvested, treated with DTT as previously described (9), and stained for HLA-A, B, C, or MICA surface antigens with PE-conjugated antibodies. (C) HEK293T cells were harvested, incubated in the presence of trypsin for 2.5 min, washed, and re-placed in 37c in aliquots. Cells were stained for both GP and HLA-I expression as before in different time points following trypsin digestion. Percent GP expression represent percent GP positive cells as compared to trypsin untreated cells; recovered cells represented same GP staining pattern as trypsin non-treated cells. Percent shielding level represent the fraction of HLA-I negative cells as compared to the fraction of the HLA-I negative cells in the trypsin non-treated cells. Results are from one representative experiment of three [(A) trypsin time titration] and two (B,C) performed. [file image_3.JPEG]

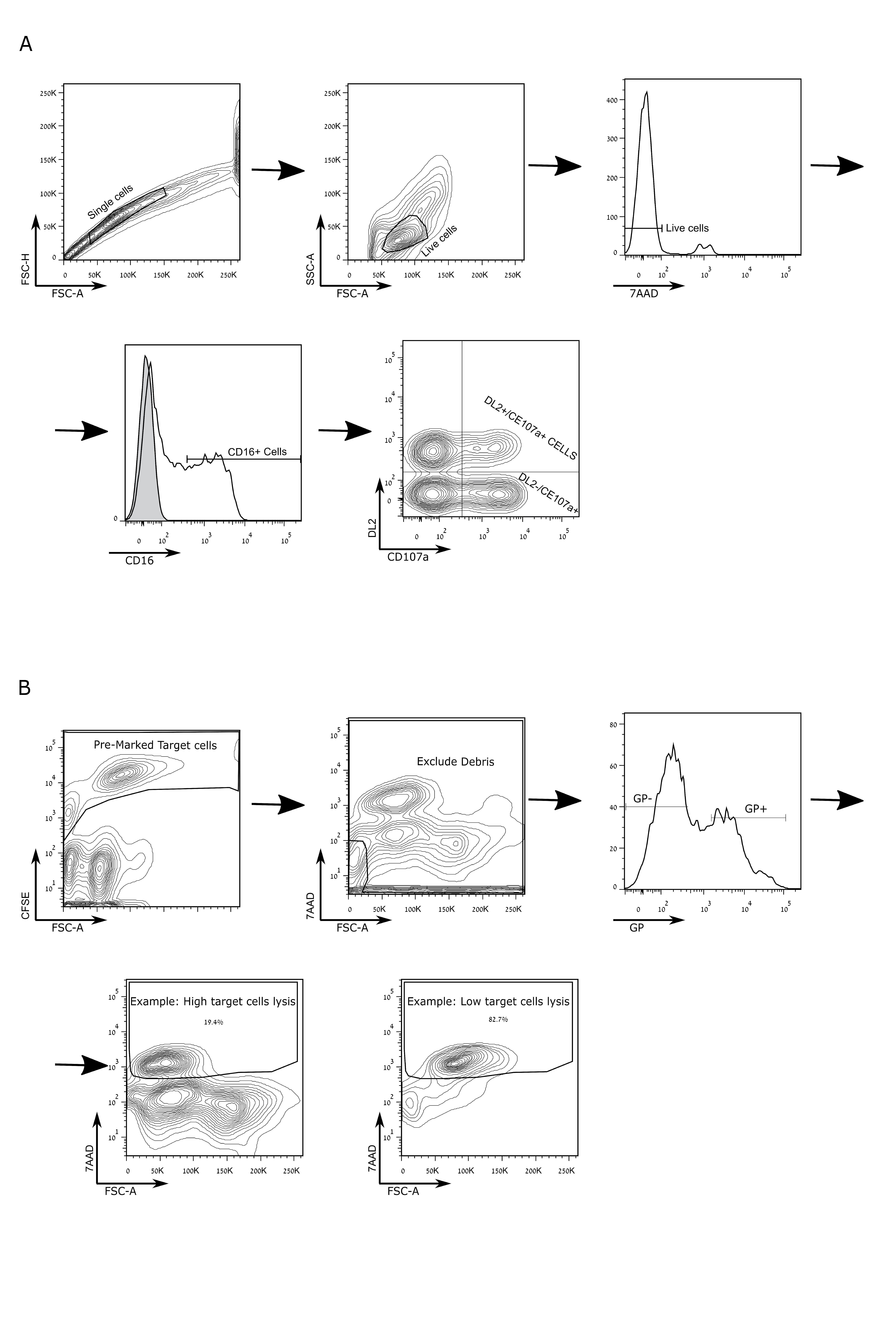

Supplement: Figure S4 — Gating strategies applied in FACS functional assays. Effector and target cells were prepared as previously described, stained, and analyzed using the following sequences: (A) degranulation assay analysis (71): single cells were gated as depicted in scheme on a FSC-H/FSC-A plot. Live pNK cells were then further gated on a SSC-A/FSC-A plot followed by gating on a 7-aminoactinomycin D (7AAD) histogram. To exclude remaining target cells, CD16-positive cells were gated and plotted on KIR2DL2/CD107a plot. (B) Specific lysis assay analysis (43): target cell population was gated on carboxyfluorescein succinimidyl ester/FSC-A plot, debris and apoptotic bodies were excluded on a 7AAD/FSC-A plot, GP+ and GP− cells were segregated by gating on a GP-allophycocyanin histogram and plotted on 7AAD/FSC-A plot to determine population specific live/dead ratio. [file image_4.JPEG]
